# Roles of RNA scaffolding in nanoscale Gag multimerization and selective protein sorting at HIV membranes

**DOI:** 10.1126/sciadv.adk8297

**Published:** 2024-02-23

**Authors:** Yachen Ying, Yantao Yang, Antony K. Chen

**Affiliations:** ^1^ Department of Biomedical Engineering, College of Future Technology, Peking University, Beijing 100871, China.; ^2^ National Biomedical Imaging Center, Peking University, Beijing 100871, China.

## Abstract

HIV-1 Gag proteins can multimerize upon the viral genomic RNA or multiple random cellular messenger RNAs to form a virus particle or a virus-like particle, respectively. To date, whether the two types of particles form via the same Gag multimerization process has remained unclarified. Using photoactivated localization microscopy to illuminate Gag organizations and dynamics at the nanoscale, here, we showed that genomic RNA mediates Gag multimerization in a more cluster-centric, cooperative, and spatiotemporally coordinated fashion, with the ability to drive dense Gag clustering dependent on its ability to act as a long-stranded scaffold not easily attainable by cellular messenger RNAs. These differences in Gag multimerization were further shown to affect downstream selective protein sorting into HIV membranes, indicating that the choice of RNA for packaging can modulate viral membrane compositions. These findings should advance the understanding of HIV assembly and further benefit the development of virus-like particle–based therapeutics.

## Teaser

RNA scaffolding is vital for HIV assembly, regulating Gag multimerization and selective protein sorting within viral membranes.

## INTRODUCTION

The HIV-1 particle is assembled through extensive interactions between the viral genomic RNA (gRNA) and viral structural protein Gag at the plasma membrane (PM). Thousands of Gag molecules are recruited to form a particle, with specific interactions between a small number of Gag and the gRNA packaging signal Ψ mediating the “fishing out” of gRNAs over the vast excess of cellular RNAs to the assembly sites at the PM, while the vast majority of Gag interacts with the longer remainder of gRNA, using gRNA as a modular scaffold to form higher-order Gag multimers while packaging gRNA (*1*–*3*). In the absence of gRNA, Gag can still multimerize upon and ultimately package a roughly equivalent mass of a nearly arbitrary collection of cellular mRNAs, forming noninfectious but morphologically normal particles [i.e., virus-like particles (VLPs)] (*4*–*6*).

Although it is well accepted that the specific Gag-Ψ interaction confers gRNA selection advantage for virus assembly, whether Gag progresses through similar multimerization pathways to package gRNA, and cellular mRNAs (referred to as cellular RNAs hereafter) has remained elusive. In vitro studies based on recombinant Gag proteins have demonstrated that diverse nucleic acids can promote efficient Gag multimerization via electrostatic Gag-RNA interaction (*7*–*10*), raising the possibility that virus and VLP formation involves similar Gag multimerization processes. On the contrary, cross-linking immunoprecipitation-based studies have reported specific nucleotide arrangements or sequences in the non-Ψ gRNA region that are preferentially bound by Gag (*2*, *11*) or host cellular proteins that can augment Gag multimerization (*12*, *13*), supporting the idea that the longer non-Ψ remainder of gRNA can also contribute to selective packaging by serving as a unique scaffold upon which Gag can efficiently multimerize. Despite these advances, the spatiotemporal organizations of Gag molecules driving the assembly of virus particles and VLPs in the native cellular context have not been systematically explored.

Standard fluorescence microscopy methods have been used to provide valuable spatiotemporal information regarding HIV-1 biogenesis, for example, the time required to complete assembly (*14*, *15*), but diffraction-limited spatial resolution (~250 nm) has hampered visualization of molecular details within the crowded assembly environment, which has a size less than 150 nm in diameter. More advanced microscopy approaches such as photoactivated localization microscopy (PALM) have allowed the characterization of nanoscale spatiotemporal organizations of individual proteins with spatial resolution approaching 20 nm in various subcellular structures, including HIV-1 assembly platforms as shown in our previous studies (*16*–*22*). This prompted the present work in which we used PALM to investigate gRNA- and cellular RNA–mediated Gag multimerization in cells. Through systematic comparisons of the nanoscale organizations and dynamics of Gag expressed in the presence or the absence of gRNA [so that cellular RNAs become predominantly packaged (*5*)], we provide evidence that Gag progresses through quite distinct pathways to multimerize around gRNA or cellular RNAs. We also demonstrated that the two processes may lead to different transmembrane protein compositions in HIV membranes.

## RESULTS

### Establishing probe transfection conditions for comparing gRNA- and cellular RNA–mediated Gag multimerization by PALM

**Fig. 1.**
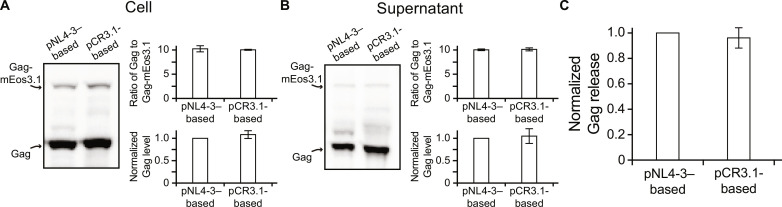
Establishing transfection conditions for comparing gRNA- and cellular RNA–mediated Gag multimerization by fluorescence imaging. COS7 cells were cotransfected with pNL4-3ΔPolΔEnv-Gag-mEos3.1 or pCR3.1-Gag-mEos3.1 in a 1:10 ratio with the respective untagged construct. A total of 2 μg of pNL4-3–based constructs and 0.4 μg of pCR3.1-based constructs were transfected to confer similar Gag expression levels in the cell and supernatant between the two transfections (see fig. S2). (**A** and **B**) Assessment of Gag expression levels. Western blot was performed with HIV–immunoglobulin (Ig) to detect Gag (55 kDa) and Gag-mEos3.1 (81 kDa) in the (A) cell and (B) supernatant at ~18 hours after transfection of the pNL4-3–based or pCR3.1-based constructs. Gag expression (normalized to the level of Gag yield by pNL4-3–based constructs) and the ratio of Gag and Gag-mEos3.1 in the cell and supernatant were calculated as described in Materials and Methods. Data represent means ± SEM of four experiments. Note that under our transfection conditions, Gag expression levels in the cell and supernatant were similar between the two expression systems, with the ratio of Gag and Gag-mEos3.1 in the cell and supernatant corresponding well to the 1:10 cotransfection ratio in both cases. (**C**) Assessment of Gag release efficiency (normalized to the level of Gag release yield by pNL4-3–based constructs). Data represent means ± SEM of four experiments.

To study gRNA- versus cellular RNA–mediated Gag multimerization by PALM, we generated a proviral pNL4-3–derivative construct named pNL4-3ΔPolΔEnv-Gag-mEos3.1 that is truncated in the polymerase and envelope genes and encodes Gag fused to the mEos3.1 photoactivatable fluorescent protein, and a cytomegalovirus (CMV) promoter–driven construct named pCR3.1-Gag-mEos3.1 that encodes Gag-mEos3.1 only (see fig. S1 for construct schematics). The tagged constructs were always transiently transfected in a 1:10 ratio with their corresponding untagged constructs in COS7 cells, a transfection condition previously shown to minimize the formation of dead-end products (*23*), with the total amounts of plasmids used for transfection always being 2 μg for the pNL4-3–based constructs and 0.4 μg for pCR3.1-based constructs to yield similar cellular and supernatant Gag levels without eliciting assembly defects caused by mEos3.1 tagging (Fig. 1 and figs. S2 to S4). These similarities were consistent with previous studies demonstrating that overexpressing Gag with and without gRNA can result in similar cellular and supernatant Gag levels as well as the amounts of Gag released (*24*). We hypothesized that under these transfection conditions, the quantities of both tagged and untagged Gag molecules within viral assembly sites before release are also similar between cells transfected with pNL4-3–based constructs (herein denoted as gRNA^+^ cells) and pCR3.1-based constructs (herein denoted as gRNA^−^ cells). Thus, any detectable differences observed by PALM should reflect distinct organizations of Gag molecules orchestrated by the different RNA molecules.

### Nanoscale organizations of gRNA- and cellular RNA–mediated Gag multimerization

**Fig. 2.**
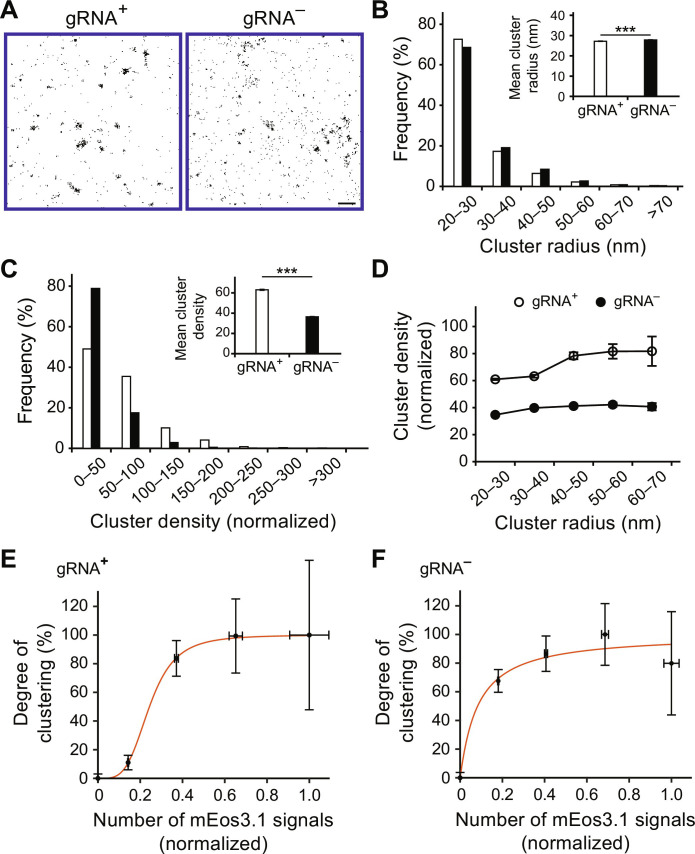
Gag forms more densely packed clusters at the PM in the presence of gRNA. (**A**) Representative PALM images of Gag in gRNA^+^ and gRNA^−^ cells. Individual spots represent single molecules. Scale bar, 500 nm. (**B**) Cluster radius distribution of Gag in gRNA^+^ cells (*n* = 7983 clusters from eight cells) and gRNA^−^ cells (*n* = 12,757 clusters from eight cells). The inset shows means ± SEM radius. (**C**) Cluster density distribution of Gag in gRNA^+^ cells (*n* = 7983 clusters from eight cells) and gRNA^−^ cells (*n* = 12,757 clusters from eight cells). For each cell, the cluster densities were normalized with respect to the mean density across the entire PM. The inset shows means ± SEM density. (**D**) Gag cluster density of gRNA^+^ and gRNA^−^ cells from (C) plotted as a function of cluster radius. (**E** and **F**) Gag cluster density from (D) was further normalized with respect to the highest mean value, and the results (i.e., degree of clustering) were plotted as a function of the number of mEos3.1 signals detected within clusters for (E) gRNA^+^ and (F) gRNA^−^ cells. The red line represents the nonlinear least-squares fitting of a four-parameter logistic regression model analogous to the Hill equation. For gRNA^+^ cells, *r*^2^ = 0.9998 and apparent cooperative index (*n*_H_) = 3.95. For gRNA^−^ cells, *r*^2^ = 0.9538 and *n*_H_ = 1.05. For (B) to (F), values were extracted from fixed-cell PALM images using a Hoshen-Kopelman–based algorithm as described in Materials and Methods. ***P < 0.001.

PALM imaging showed that a punctate staining pattern, indicative of assembling Gag complexes, was readily observed across the PM of both gRNA^+^ and gRNA^−^ cells (Fig. 2A). Cluster analysis revealed similar abilities of Gag to form sizable clusters in both cells, with >99% of the clusters representing assembling clusters having a radius less than the radius of a completely assembled virion (70 nm) (Fig. 2B and fig. S5). Despite this similarity, the Gag nanoscale organizations in the two cellular contexts were quite different. Specifically, a nearly twofold greater number of Gag clusters were detected in the gRNA^−^ cells, while in gRNA^+^ cells, Gag clusters were nearly two times more densely packed than those in gRNA^−^ cells for all cluster sizes measured (Fig. 2, C and D; fig. S6; and table S1). In addition, Gag cluster density increased with increasing cluster size in a sigmoidal fashion in gRNA^+^ cells, as shown in our previous finding (*19*), with an inflection point of ~45.5 nm separating the initial exponential phase and the late asymptotic phase (fig. S7). In contrast, Gag cluster density appears fairly constant during assembly in gRNA^−^ cells. Similar results were obtained when analogous experiments were performed in HeLa cells [figs. S8 (A to D) to S10]. The differences between the two Gag nanoscale organizations were not due to the different expression systems used, as an assembly-competent Gag mutant in which the major RNA binding (nucleocapsid) domain is replaced with an isoleucine zipper motif (Gag_ZiL_-mEos3.1; fig. S1) expressed from pNL4-3–based and pCR3.1-based constructs (coexpressed with the respective untagged constructs in a 1:10 ratio) could form similarly dense clusters and exhibiting similar linear cluster density versus cluster size relationships (figs. S11 and S12), which reflected RNA-independent Gag_ZiL_ multimerization (*19*, *25*). Moreover, an assembly-defective Gag mutant lacking the capsid C-terminal domain (CTD) responsible for Gag-Gag interactions (Gag-ΔCA_CTD_-mEos3.1; fig. S1) expressed from the two systems (coexpressed with Gag-ΔCA_CTD_ in a 1:10 ratio) could both result in little, if any, sizeable clusters and particle formation (fig. S11). Therefore, Gag forms less densely packed clusters when multimerizing upon cellular RNAs.

### Different levels of conformity to cooperative Gag-RNA interaction between gRNA- and cellular RNA–mediated Gag multimerization

In vitro studies have indicated RNA packaging by Gag as a cooperative process that can be modeled by the Hill equation; that is, RNA packaging is driven by Gag binding with one another and with RNA (*9*, *10*). This raises the question of whether the observed differences in Gag clustering between gRNA^+^ and gRNA^−^ cells were due to differences in cooperative binding between Gag and gRNA versus cellular RNAs. To test this, we normalized Gag-mEos3.1 cluster densities (from Fig. 2D) with respect to the highest mean value to obtain the degree of mEos3.1 clustering, which should reflect the degree of RNA packaging by Gag (*9*, *10*). The results were plotted as a function of the number of mEos3.1 signals detected for each cluster (see Materials and Methods). It was found that the degree of clustering as a function of the number of mEos3.1 signal increases sigmoidally in gRNA^+^ cells and asymptotically in gRNA^−^ cells (Fig. 2, E and F). Both plots could be fitted, with high degrees of confidence (*r*^2^ > 0.95), to a four-parameter logistic regression model analogous to the Hill equation (see Materials and Methods). Notably, the exponent (i.e., the apparent cooperative index, *n*_H_) associated with the mEos3.1 signal required for completing Gag assembly was 3.95 in gRNA^+^ cells compared to 1.05 in gRNA^−^ cells, suggesting that Gag multimerization is much more cooperative in the presence of gRNA. Similar results were also observed when analogous analyses were performed on Gag clusters identified in HeLa cells (fig. S8, E and F). Therefore, in contrast to in vitro Gag-RNA binding results suggesting that any packageable RNA may drive Gag multimerization via similar cooperative processes under physiologically relevant salt concentrations (*9*), our in cellulo results suggest that cooperative Gag-RNA interaction is a more prominent feature of gRNA packaging in the bona fide assembly environment.

### Nanoscale dynamics of gRNA- and cellular RNA–mediated Gag multimerization

Since gRNAs are considerably longer than cellular RNAs packaged, yet a gRNA-packaging particle and a cellular RNA–packaging particle contain roughly the same amounts of ribonucleotides (*5*, *6*), we speculated that gRNA-mediated Gag multimerization is more efficient and cooperative because gRNA, which presumably exists as a dimer through Gag multimerization (*1*, *26*–*28*), could act as a single long-stranded continuous scaffold to bind Gag molecules and stabilize Gag binding. By contrast, when cellular RNAs are instead packaged, more copies of the shorter and different cellular RNAs can behave as separate scaffolds, which independently bind different subsets of Gag molecules to form different Gag-RNA complexes that display less coordinated and perhaps conflicting behaviors, which may hamper dense Gag clustering. To test this possibility, we studied the mobility of Gag molecules at the PM of gRNA^+^ and gRNA^−^ cells using single-particle tracking PALM. This was followed by Bayesian model selection to hidden Markov modeling (HMM-Bayes) (*29*) and time-correlated PALM (tcPALM) (*30*) analyses to identify any changes in single-molecule Gag motions (see Materials and Methods).

**Fig. 3.**
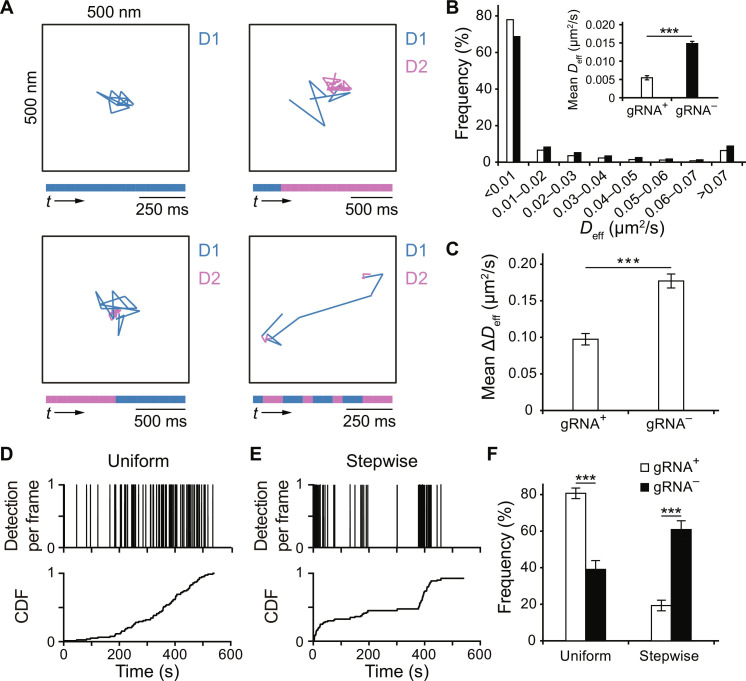
Gag exhibits more stable dynamics in the presence of gRNA. (**A** and **B**) Analysis of Gag dynamics at the PM of gRNA^+^ and gRNA^−^ cells by HMM-Bayes. (A) Representative full-track movements of Gag. Different diffusion states, denoted as D1 and D2, were represented by different colors (i.e., blue and pink), and the temporal sequence of motion states was shown as a colored bar under each trajectory. (B) Diffusion coefficient (*D*_eff_) distribution of Gag in gRNA^+^ cells (*n* = 5891 motions from 21 cells) and gRNA^−^ cells (*n* = 8957 motions from 18 cells). The inset shows means ± SEM *D*_eff_. (**C**) The magnitude of motion switching (Δ*D*_eff_) between different states. Data represent means ± SEM from *n* = 120 trajectories from 21 gRNA^+^ cells and *n* = 298 trajectories from 18 gRNA^−^ cells. (**D** to **F**) Analysis of Gag dynamics at the PM of gRNA^+^ and gRNA^−^ cells by tcPALM. (D and E) Representative (D) uniform and (E) stepwise Gag tcPALM profiles as well as their associated cumulative distribution functions (CDFs). (F) Percentage of Gag clusters having uniform or stepwise tcPALM profiles. Data represent means ± SEM of 21 gRNA^+^ cells and 18 gRNA^−^ cells. ***P < 0.001).

HMM-Bayes revealed that while most Gag molecules (>99%) at the PM of the two cells displayed diffusive motion, Gag could exhibit more rapid movement and increased motion switching in the absence of gRNA (Fig. 3, A to C), as expected if the different cellular RNAs packaged behave as separate and uncollaborative entities compared to a packaged single gRNA dimer. Supporting this view, tcPALM identified more sporadic appearances of high-frequency Gag signal detections in gRNA^−^ cells (Fig. 3, D to F), reflecting more formation of sizeable Gag subcomplexes or intermediates via scaffolding by different cellular RNAs. Collectively, these findings support the view that cellular RNAs, by virtue of being “unlinked” with one another and thus behaving as separate entities, cannot collectively provide a large binding surface for efficient and robust Gag multimerization as can do so by a single gRNA dimer. To test this idea further, we generated a miniature pNL4-3–derivative construct (pNL4-3 mini–Gag–mEos3.1) that expresses a short, 3.5 kb in length packageable gRNA (*31*) termed gRNA mini (figs. S1 and S13). It was found that Gag-mEos3.1 forms less densely packed clusters and exhibits less conformity to cooperative Gag-RNA interactions in cells cotransfected with this miniature construct in a 1:10 ratio with the untagged miniature construct (pNL4-3 mini) compared with gRNA-mediated Gag multimerization (fig. S14). This suggests that the unique size of gRNA confers its ability to act as a scaffold for dense clustering of Gag.

### The choice of RNA for packaging and protein partitioning into HIV membranes

To date, much evidence has shown that Gag multimerization can actively remodel PM organization, forming a unique HIV membrane that exhibits a liquid-ordered (Lo) structure enriched in sphingomyelins, glycosphingolipids, cholesterol, and phosphatidylinositol 4,5-bisphosphate lipid [PI(4,5)P_2_] (*32*–*36*). Moreover, it has been demonstrated that the Gag multimerization–mediated lipid reorganization may also serve as a driving force for the selective recruitment of certain PM proteins having affinities for the ordered lipid environment (*32*), providing insights into why certain proteins are usurped by the virus to evade immune surveillance. Despite these advances, whether the choice of RNA for packaging can influence the selective lipid and protein sorting processes at the HIV membrane has not been explored. Given our findings that Gag multimerization displays quite distinct nanoscale spatiotemporal organizations and dynamics between gRNA and cellular RNA packaging, we hypothesized that these differences may have important biological consequences for the unique HIV membrane composition.

To test the above idea, we generated pNL4-3–based and pCR3.1-based Gag-mCherry constructs and transfected them (in a 1:10 ratio to the corresponding untagged constructs) in COS7 and HeLa cells expressing an Lo phase marker glycosyl phosphatidylinositol–anchored enhanced green fluorescent protein (EGFP-GPI), a liquid-disordered phase marker geranylgeranylated EGFP (EGFP-GG), or murine leukemia virus envelope glycoprotein fused with EGFP (MLV-Env-EGFP), a transmembrane protein that harbors EGFP in the hypervariable region in the extracellular domain (figs. S1 and S15E) that was shown to avoid functional defects (*37*–*40*) and displays a preference to localize to Lo phase due to its palmitoylated cytoplasmic tail (*41*, *42*). Since these proteins have no known specific interactions with Gag or RNA, we hypothesized that any detectable differences in their distributions across the HIV membrane should reflect different assembly environments created by the different RNAs. At ~18 hours after transfection, COS7 and HeLa cells were fixed, and images were acquired in the mCherry and EGFP channels by total internal reflection fluorescence (TIRF) microscopy, and the extent of enrichment or depletion of these proteins in the assembly sites (marked by diffraction-limited Gag-mCherry fluorescence spots) was computed on the basis of their signal intensities at assembly sites relative to their average intensities over the PM (see Materials and Methods).

**Fig. 4.**
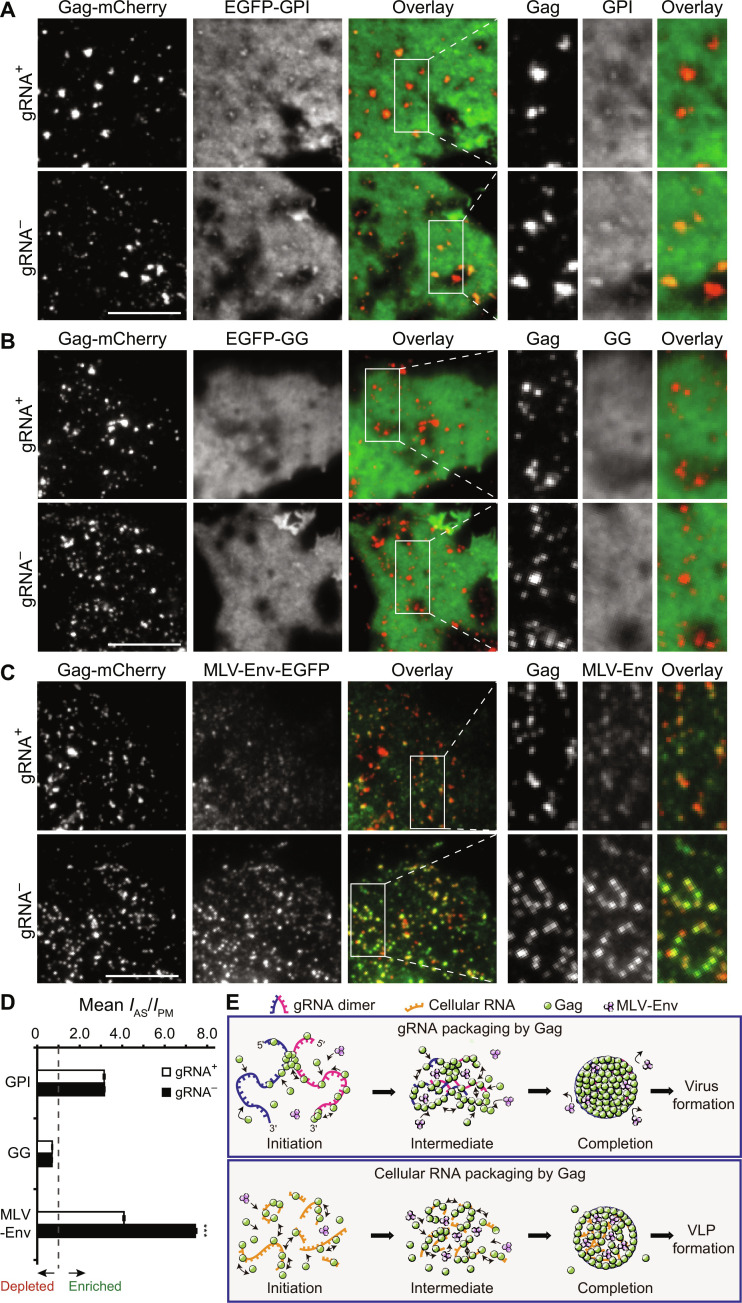
PM proteins exhibit differential partitioning into cellular RNA–mediated versus gRNA-mediated particle assembly sites in COS7 cells. (**A** to **C**) Representative TIRF images of Gag-mCherry and (A) EGFP-GPI, (B) EGFP-GG, and (C) MLV-Env-EGFP at the PM of gRNA^+^ and gRNA^−^ cells at ~18 hours after transfection. The panels on the right are magnified images of the boxed areas in the images. Scale bars, 10 μm. (**D**) Extents of enrichment and depletion of indicated PM proteins at the Gag assembly sites (means ± SEM, *I*_AS_/*I*_PM_) in gRNA^+^ and gRNA^−^ cells (see Materials and Methods). For EGFP-GPI, *n* = 3769 assembly sites from 29 gRNA^+^ cells and *n* = 6919 assembly sites from 25 gRNA^−^ cells. For EGFP-GG, *n* = 4203 assembly sites from 27 gRNA^+^ cells and *n* = 6103 assembly sites from 23 gRNA^−^ cells. For MLV-Env-EGFP, *n* = 6328 assembly sites from 43 gRNA^+^ cells and *n* = 7445 assembly sites from 39 gRNA^−^ cells. ***P < 0.001). (**E**) Schematic model illustrating gRNA- and cellular RNA–mediated Gag multimerization and MLV-Env partitioning at assembly sites. The compact and crowded assembly environment created by gRNA accommodates limited MLV-Env molecules, whereas the less compact and more fluid environment created by cellular RNA is more permissive for MLV-Env incorporation.

It was found that all three EGFP-fusion proteins could exhibit the expected partitioning preferences at both gRNA- and cellular RNA–associated assembly sites in both COS7 and HeLa cells (Fig. 4, A to C, and fig. S15, A to C). Specifically, the two lipid markers EGFP-GPI and EGFP-GG exhibited similar extents of enrichment and depletion, respectively, between the two types of Gag assembly sites, suggesting that gRNA- and cellular RNA–mediated Gag multimerization, despite having different Gag cluster densities, can result in similar PM lipid organizations. In contrast, MLV-Env-EGFP exhibits different spatial organizations between the two Gag multimerization processes. Specifically, while MLV-Env-EGFP was enriched in Gag assembly sites under both conditions, its localization was lower in assembly sites scaffolded by gRNA (Fig. 4D and fig. S15D). Thus, gRNA- and cellular RNA–mediated Gag multimerization can result in similar lipid organizations but different MLV-Env-EGFP distributions at viral assembly sites. To test this further, we generated pNL4-3–based and pCR3.1-based constructs encoding the RNA binding defective mutant Gag_ZiL_ tagged with mCherry (Gag_ZiL_-mCherry). Gag_ZiL_-mCherry expressed from both constructs (coexpressed with the respective untagged constructs in a 1:10 ratio) could lead to similar MLV-Env-EGFP enrichments, as expected if Gag multimerization can drive selective recruitment of MLV-Env-EGFP through reorganization of the lipid distribution independent of RNA (figs. S16 and S17). Notably, the extents of Gag_ZiL_ multimerization–mediated MLV-Env-EGFP enrichments were also comparable to the MLV-Env-EGFP enrichment observed with gRNA-mediated Gag multimerization but less than that observed with cellular RNA–mediated Gag multimerization. Given that Gag_ZiL_ clusters, on average, have cluster densities similar to the average density of Gag clusters packaging gRNA but higher than that of Gag clusters packaging cellular RNAs (compare Fig. 2C and fig. S12C), we concluded that MLV-Env-EGFP cannot be recruited into the highly compacted gRNA-associated assembly environment as effectively as the more permeable cellular RNA–associated assembly environment.

## DISCUSSION

In summary, we present that HIV-1 Gag undergoes distinguishable multimerization pathways to package gRNA and cellular RNAs at the nanoscale. Moreover, the differences in Gag multimerization between the two packaging processes can have functional consequences for downstream viral processes in terms of recruiting additional PM proteins into HIV membranes. Specific findings are as follows: (i) Although the levels of Gag expression were comparable (Fig. 1), gRNA-associated Gag clusters and cellular RNA–associated Gag clusters have different quantities and Gag densities (Fig. 2); (ii) compared with gRNA, cellular RNAs are packaged in a less cluster-centric and less cooperative manner, with more sporadic appearances of Gag intermediates exhibiting uncoordinated and perhaps conflicting movements (Figs. 2 and 3). These differences reflect the ability of the dimer-forming gRNA to form a long-stranded, continuous scaffold not attainable by the multiple random cellular RNAs; (iii) while Gag multimerization mediates similar degrees of lipid partitioning (as indicated by EGFP-GPI enrichment and EGFP-GG depletion) at HIV membranes between gRNA and cellular RNA packaging, the Lo phase preferring MLV-Env-EGFP, which exhibits a preference to partition into HIV membranes due to its palmitoylated cytoplasmic tail, is less enriched in the HIV membrane of the gRNA-associated assembly environment (Fig. 4). We should emphasize that the experiments were performed under conditions where Gag molecules are overexpressed at similar levels between gRNA^+^ and gRNA^−^ cells. Moreover, early assembly events below the 20-nm length scale (the spatial resolution limitation of PALM), including the formation of Gag assembly precursors and the subsequent growth of these precursors into a visualizable cluster (*10*, *43*), as well as their potential contributions to HIV membrane organizations, were not detected. However, the methodologies presented in this study may potentially be expanded to explore these important aspects in future studies, deepening our understanding of the overall virus and VLP assembly processes.

Our results support a model wherein gRNA, which is presumably dimeric following the initiation of Gag multimerization (*1*, *26*–*28*), may serve as a long-stranded modular scaffold to promote Gag multimerization by allowing Gag molecules to interact not only with one another on the same gRNA monomer but also with those bound to the other gRNA monomer via a cooperative process (Fig. 4E). The multiple packaged cellular RNAs, on the other hand, function as separate scaffolds that can each only support the multimerization of a subset of Gag molecules, with the cellular RNA–Gag complexes so formed not capable of multimerizing further into larger complexes as efficiently and robustly as when all Gag molecules are connected via a single long-stranded gRNA scaffold. In this context, a transmembrane protein (e.g., MLV-Env-EGFP) can exhibit reduced entry into the viral assembly site in the presence of gRNA because the densely packed Gag assembly complexes cause steric hindrance to the protein’s cytoplasmic tail.

Currently, the mechanism underlying the selective packaging of the HIV-1 viral genome in the presence of a vast excess of cellular RNAs has remained elusive. Our findings that HIV-1 Gag assembly occurs through a more cluster-centric and a more cooperative multimerization pathway in the presence of gRNA should lend strong support to the hypothesis that the HIV-1 gRNA nucleates assembly more efficiently than cellular RNAs (*1*, *10*, *24*). In addition, the findings raise the possibility that released particles encapsidating gRNAs or cellular RNAs may exhibit different Gag compositions and organizations. Moreover, the observed differential enrichment of MLV-Env-EGFP in the different RNA-mediated Gag multimerization environments should represent a previously undescribed phenomenon that might add value to our current understanding of how the organizations of transmembrane proteins of both viral and cellular origins are regulated by HIV-1 Gag. Specifically, it suggests that while a transmembrane protein can be specifically recruited into an HIV-1 assembly site due to lipid-based partitioning, its spatial distribution within the assembly site can still be further fine-tuned by steric hindrance imposed by gRNA-mediated Gag clustering on its cytoplasmic tail. Given that MLV-Env has a short cytoplasmic tail (36 amino acids in length) relative to other transmembrane proteins, the proposed gRNA-mediated steric hindrance mechanism may also have important implications for understanding why certain proteins with larger cytoplasmic tails such as CD4, despite exhibiting Lo phase–preferring characteristics, cannot be effectively recruited to HIV membranes (*44*–*46*). A similar steric-based regulation may also explain why the HIV-1 envelope protein, despite the potential specific interactions between Gag and the protein’s large cytoplasmic tail (*47*, *48*), cannot be recruited as effectively as MLV-Env into the HIV-1 membrane (*49*, *50*).

The findings that Gag multimerization in the absence of gRNA can result in increased MLV-Env-EGFP recruitment should have important implications in vaccine development involving the engineering of HIV Gag-based VLPs functionalized with viral or nonviral transmembrane proteins as antigens (*51*). For example, beyond offering advantages such as increased safety and ease of manufacturing, our findings suggest that VLP-based vaccines might potentially offer greater therapeutic potency compared to conventional live-attenuated and inactivated vaccines due to the ability to accommodate a greater number of antigens owing to the absence of the viral genome. In addition, the findings may potentially inspire new VLP designs by raising the possibility of removing nonessential components of a candidate antigen’s cytoplasmic tail to achieve a greater antigen density on the particle surface (*52*).

## MATERIALS AND METHODS

### Cell culture

COS7 and HeLa cell lines (American Type Culture Collection), as well as HeLa cells stably expressing EGFP-GPI, EGFP-GG and MLV-Env-EGFP, were cultured in Dulbecco’s modified Eagle’s medium (Mediatech), supplemented with 10% (v/v) fetal bovine serum (PAN-Biotech), 1× GlutaMAX (Thermo Fisher Scientific) at 37°C, 5% (v/v) CO_2_, and 90% relative humidity. All experiments were performed with cells at passage numbers between 5 and 25.

### Plasmid construction

#### PNL4-3–based constructs

pNL4-3ΔPolΔEnv and pNL4-3ΔPolΔEnv-Gag_ZiL_ have been described previously (*19*, *21*). pNL4-3ΔPolΔEnv-Gag-ΔCA_CTD_ was created by overlap extension polymerase chain reaction (PCR) using pNL4-3ΔPolΔEnv as the template, followed by insertion of the corresponding PCR products into the Bss HII/Eco RI–digested pNL4-3ΔPolΔEnv vector. pNL4-3ΔPolΔEnv-Gag-mEos3.1 was constructed by overlap extension PCR using mEos3.1-N1 (*53*) (a gift of P. Xu, Chinese Academy of Sciences, Beijing, China) and pNL4-3ΔPolΔEnv as templates. The resulting product was then inserted into the Bss HII/Spe I–digested pNL4-3ΔPolΔEnv vector. pNL4-3ΔPolΔEnv-Gag-ΔCA_CTD_-mEos3.1 and pNL4-3ΔPolΔEnv-Gag_ZiL_-mEos3.1 were created by inserting the Spe I/Eco RI–digested pNL4-3ΔPolΔEnv-Gag-ΔCA_CTD_ fragment and the Spe I/Eco RI–digested pNL4-3ΔPolΔEnv-Gag_ZiL_ fragment into the pNL4-3ΔPolΔEnv-Gag-mEos3.1 vector, respectively. The untagged and mEos3.1-tagged miniature Ψ-containing pNL4-3–derivative constructs (i.e., pNL4-3 mini and pNL4-3 mini–Gag–mEos3.1) were generated by overlap extension PCR using pNL4-3ΔPolΔEnv as template, followed by insertion of the resulting products into the Spe I/Nco I–digested pNL4-3ΔPolΔEnv vector and pNL4-3ΔPolΔEnv-Gag-mEos3.1 vector, respectively. pNL4-3ΔPolΔEnv-Gag-mCherry was constructed by overlap extension PCR using pmCherry-N1 (Clontech) and pNL4-3ΔPolΔEnv as templates. The resulting product was then inserted into the Bss HII/Spe I–digested pNL4-3ΔPolΔEnv vector. pNL4-3ΔPolΔEnv-Gag_ZiL_-mCherry was created by inserting the Spe I/Eco RI–digested pNL4-3ΔPolΔEnv-Gag_ZiL_ fragment into the pNL4-3ΔPolΔEnv-Gag-mEos3.1 vector. See table S2 for the PCR primers used.

#### CMV-driven expression plasmids encoding HIV-1 Gag or Gag variants

pCR3.1-Gag (*54*) was a gift of S. Simons (Rockefeller University, New York, NY). pCR3.1-Gag_ZiL_ was generated by PCR amplification of the isoleucine zipper coding region from pNL4-3ΔPolΔEnv-Gag_ZiL._ The resulting blunt end fragment was then inserted into pCR3.1-Gag vector generated by PCR. pCR3.1-Gag-mEos3.1 was generated by overlap extension PCR using mEos3.1-N1 and pCR3.1-Gag as templates. The resulting product was then inserted into the Eco RI/Age I–digested pCR3.1-Gag vector. pCR3.1-Gag_ZiL_-mEos3.1 was generated by inserting the Eco RI/Age I–digested pCR3.1-Gag-mEos3.1 fragment into the pCR3.1-Gag_ZiL_ vector digested with the same enzymes. pCR3.1-Gag-ΔCA_CTD_ and pCR3.1-Gag-ΔCA_CTD_-mEos3.1 were generated by overlap extension PCR using pCR3.1-Gag and pCR3.1-Gag-mEos3.1 as templates, respectively, followed by insertion of the resulting products into the Sma I/Not I–digested pCR3.1-Gag vector. pCR3.1-Gag-mCherry was generated by overlap extension PCR using pmCherry-N1 and pCR3.1-Gag as templates. The resulting product was then inserted into the Eco RI/Age I–digested pCR3.1-Gag vector. pCR3.1-Gag_ZiL_-mCherry was generated by inserting the Eco RI/Age I–digested pCR3.1-Gag-mCherry fragment into the pCR3.1-Gag_ZiL_ vector digested with the same enzymes. See table S3 for the PCR primers used.

To create CMV-driven Tat and Rev expressing helper constructs, the Tat and Rev gene sequences were first PCR amplified from pCV1 (National Institutes of Health AIDS Research and Reference Reagent Program, ARP-303) using the following primers: Tat, 5′-GTGGTGGAATTCCAAGAAATGGAGCCAGTAGATCCTAGAC-3′ (forward) and 5′-TCCTTAGCGGCCGCACAGCACTATTCCTTCGGGCCTGTCGGG-3′ (reverse); Rev, 5′-GTGGTGGAATTCCAAGAAATGGCAGGAAGAAGCGGAGACAG-3′ (forward) and 5′-TCCTTAGCGGCCGCACAGCACTATTCTTTAGCTCCTGACTC-3′ (reverse). The PCR products were then digested with Eco RI and Not I, followed by insertion of the digested fragments into the pCR3.1 vector digested with the same enzymes.

Plasmids encoding EGFP-GPI and MLV-Env-EGFP were gifts from P. Sengupta (Janelia Research Campus, Ashburn, VA) (*32*) and M. C. Johnson (University of Missouri, Columbia, MO) (*40*), respectively. EGFP-GG was generated using overlap extension PCR based on sequences (see table S4) described previously (*55*).

### Transfection

pNL4-3– or pCR3.1-based constructs were transiently transfected into cells using FuGene 6 (Promega) following the manufacturer’s protocols when cells reach 50 to 70% confluency. Unless otherwise noted, a total amount of 2 μg of pNL4-3–based constructs or 0.4 μg of pCR3.1-based constructs were transfected for every 7 × 10^5^ cells for all studies, since these transfection conditions could yield similar total supernatant Gag and cellular Gag levels as assessed by Western blot analysis. Moreover, the mEos3.1-tagged or mCherry-tagged constructs were cotransfected with the corresponding untagged constructs in a 1:10 ratio to rescue the assembly defects seen in cells transfected with fluorescent protein (FP)–tagged constructs only (*23*). For studies that investigate Gag nanoscale organization derived from pNL4-3 mini constructs, 250 ng of Tat and 250 ng of Rev helper constructs were also transfected. For studies that investigate lipid and PM protein partitioning at Gag assembly sites in COS7 cells, plasmids encoding EGFP-GPI (100 ng), EGFP-GG (50 ng), or MLV-Env-EGFP (150 ng) were also transfected.

### Establishment of cell lines stably expressing EGFP-GPI, EGFP-GG, and MLV-Env-EGFP

HeLa cells were transfected with the plasmids expressing EGFP-GPI, EGFP-GG, or MLV-Env-EGFP using FuGene HD (Promega). Twenty-four hours following the transfection, cells were cultured in media containing geneticin (0.8 to 5 mg/ml) for 3 weeks. Single colonies were isolated and maintained in the absence of any antibiotics. Cells that were positive for the plasmid constructs were identified by EGFP fluorescence.

### Gag particle collection

Gag particles were collected as previously described (*19*, *56*). Briefly, the culture supernatant of cells harvested at 18 hours after transfection of the Gag-expressing constructs was collected and centrifuged at 1000*g* for 10 min, followed by removal of cell debris and large aggregates using a 0.45-μm syringe filter (Pall Corporation). The eluent was then subjected to Western blot analysis or analysis of Gag colocalizations with gRNA or gRNA mini (expressed from pNL4-3 mini plus pNL4-3 mini–Gag–mEos3.1) in particles as described below.

### Western blot analysis of Gag release efficiency

After collection of the supernatant eluent as described above, Gag release efficiency was assessed by Western blot as previously described (*19*, *20*, *56*). Briefly, 2 μl of Dynabeads 280 streptavidin (Life Technologies), precleaned twice with 1× phosphate-buffered saline (PBS), was added to every milliliters of the eluent to assist visualization of the pellet after ultracentrifugation at 100,000*g* for 1 hour. The pellet (containing the particles and beads) was lysed in lysis buffer [0.5% (v/v) Triton X-100, 50 mM tris-HCl (pH 7.5), and 300 mM NaCl] supplemented with 1% (v/v) protease inhibitor cocktail (Sigma-Aldrich) for 30 min at 4°C before centrifugation at 21,000*g* for 30 min to separate the particle lysates from the beads and cell membrane debris. To collect cell lysates, the cells following supernatant removal were washed once in cold 1× PBS and then lysed directly in lysis buffer containing 1% (v/v) protease inhibitor cocktail. The presence of Gag in the supernatant and cell lysates was then analyzed by SDS–polyacrylamide gel electrophoresis on 10% bis-tris gels (Life Technologies) and transferred to Immobilon-P membranes (Millipore). After immunoblotting using HIV–immunoglobulin (Ig) (pooled Ig from HIV-1–infected patients, obtained from the National Institutes of Health AIDS Research and Reference Reagent Program), Gag levels in the supernatant and cells were determined via densitometry analysis of the resulting Western blot images using the Fiji software (*57*). Gag release efficiency was calculated as the ratio of supernatant Gag to total (supernatant plus cellular) Gag.

### Transmission electron microscopy

Cells were fixed with 2% (v/v) glutaraldehyde and 2% (w/v) paraformaldehyde in 0.1 M phosphate buffer [50 mM Na_2_HPO_4_ and 50 mM NaH_2_PO_4_ (pH 7.4)] for 1 hour at room temperature and then postfixed in 2% (w/v) osmium tetroxide and 1.5% (w/v) potassium ferrocyanide for 1 hour. After rinsing several times in distilled water, the cells were stained in 1% uranyl acetate at 4°C overnight. The samples were washed again in distilled water and dehydrated in a graded ethanol series and embedded in Embed 812 resin (Electron Microscopy Sciences). Ultrathin (70 nm) sections were cut using an ultramicrotome (Leica Microsystem, UC7) and collected on copper grids with a single slot, stained with uranyl acetate and lead citrate. The sections were observed under a JEM-1400Flash electron microscope (JEOL) operating at 80 kV equipped with a 20-megapixel XAROSA digital camera (EMSIS).

### Fluorescence microscopy

All fluorescence microscopy experiments were performed on an Olympus IX 83 motorized inverted fluorescence microscope equipped with the CellTIRF-4Line system, a back-illuminated electron-multiplying charge-coupled device camera (Andor), Sutter excitation and emission filter wheels under the control of the CellSens Dimension software. Wide-field fluorescence microscopy images were acquired using a 100× UPlanSApo 1.4 numerical aperture (NA) objective lens, an EXFO X-Cite Series 120 light source, an Olympus MT20 filter set for 4′,6-diamidino-2-phenylindole, EGFP, and Tetramethylrhodamine (TAMRA), and a Chroma filter set (ET620/60x, ET700/75m, and T660lpxr) for Qusar670. Three-dimensional (3D) image stacks were acquired with 0.25-μm increments in the *z* direction, and the image stacks were processed using AutoQuant deconvolution software (Media Cybernetics) and then by Fiji to create a maximum intensity projection image. TIRF imaging and PALM imaging were performed using a 100× 1.46 NA total internal reflection objective with 405-nm (100 mW), 488-nm (150 mW), 561-nm (150 mW), and 640-nm (140 mW) excitation lasers.

### PALM sample preparation and image acquisition

Cells were grown on fibronectin-coated, 25-mm #1.5 round glass coverslips (Warner Instruments) cleaned as previously described (*58*). At ~18 hours after transfection, cells were subjected to fixed-cell or live-cell PALM imaging following previously established procedures (*19*). Briefly, for the former case, cells were fixed with 4% (w/v) paraformaldehyde and 0.1% (w/v) glutaraldehyde in 1× PBS at room temperature for 20 min, followed by three washes with 1× PBS containing 50 mM glycine for 10 min and then one wash with 1× PBS. Thereafter, cells were imaged in 1× PBS containing Tetraspek beads (Life Technologies) to correct for *x*-*y* drift at room temperature. In the latter case, cells were placed in phenol red–free Dulbecco’s modified Eagle’s medium containing 25 mM Hepes and 1% (v/v) fetal bovine serum and then imaged at 37°C. All images were obtained by spontaneous photoconversion of mEos3.1 probes, with intermittent applications of 405-nm activation light to recover additional peaks. Time-lapse images were acquired at 10 frames/s and 20 frames/s for fixed-cell PALM and live-cell PALM experiments, respectively.

### PALM cluster analysis

Cluster analysis was performed following the procedures described previously (*19*, *20*). Briefly, peaks were localized using a program written in Interactive Data Language (Research Systems Inc.) (*18*), and those with localization precision less than or equal to 25 nm were used for further processing. Subsequently, a custom-written MATLAB algorithm was used to replace peaks appearing in consecutive frames within a radius of three times the upper limit of the localization precision (i.e., 25 nm) with a single peak whose position coordinates were determined as a weighted average of the original ones. Clusters were identified from the composite PALM image using a custom-written Hoshen-Kopelman algorithm–based MATLAB code, which groups an mEos3.1 signal and its shared neighbors as one cluster and computes the cluster’s convex hull. The radius of a circle of equivalent area as the convex hull was used as the estimate of cluster radius. Cluster density was calculated by dividing the total number of mEos3.1 signals within the convex hull by the area of the convex hull. The resulting value obtained for each cluster was then normalized with respect to the mean density of mEos3.1 over the entire PM of the cell. Only clusters with a radius less than or equal to 150 nm and density greater than three times the mean density of mEos3.1 over the PM were considered as assembling platforms for analysis. For studies that compared gRNA-associated Gag clusters and gRNA mini-associated Gag clusters (expressed from pNL4-3 mini and pNL4-3 mini–Gag–mEos3.1), fluorescence in situ hybridization (FISH) was performed to localize gRNA or gRNA mini (described below) after PALM sample preparation. Before PALM data acquisition as described above, a TIRF image in the FISH channel was acquired and used to generate a binary mask. On the basis of the *x*-*y* coordinates of TetraSpeck beads appearing in both channels, the mask was aligned to the composite mEos3.1 PALM image. The mEos3.1 signals enclosed by the mask were isolated for further analysis of cluster radius and density as described above.

### Assessment of apparent cooperativity

The cluster densities as determined by PALM cluster analysis described above were determined for each cluster size range and were normalized to the highest mean value to obtain measurements of the degree of clustering (in percentage). The results were then plotted as a function of the number of Gag signals detected with the same cluster size range. Using the nonlinear least-squares solver lsqcurvefit available in MATLAB, the data were fitted to the following equation analogous to the Hill cooperative model as previously described (*9*):

$$Y\left(x\right)=\frac{1}{1+{\left(\frac{K}{x}\right)}^{n_{H}}}$$

where *Y* represents degree of clustering (in percentage), *x* represents normalized number of mEos3.1 signals, *n*_H_ is the apparent cooperativity index, and *K* is the normalized number of mEos3.1 signals that corresponds to 50% degree of clustering.

### tcPALM analysis and profile classification

Assembly dynamics of Gag clusters was characterized using tcPALM as described previously (*30*, *59*), with some modifications. Briefly, the single-molecule localizations of mEos3.1-labeled molecules from live-cell PALM time-lapse images were first determined as described above in fixed-cell PALM analysis and used for the reconstruction of a localization density map. Ten to 15 high-density nanoclusters were randomly selected per cell, and a number of detections in a nanocluster were recorded to yield cumulative distribution function that can be used as the basis to characterize the dynamics of cluster formation. A step increase with the magnitude greater than 20% of the total number of detections identified, using a stepfinder algorithm (*60*), was used as the threshold to determine whether a cluster grew in a stepwise or a uniform fashion.

### HMM-Bayes analysis

Live-cell PALM time-lapse images were analyzed to identify the trajectories of mEos3.1-labeled molecules by the TrackMate plugin available in Fiji following procedures similar to those previously described (*19*, *20*). Briefly, the localization of individual mEos3.1 signals was determined by the Laplacian of Gaussian detector (estimated blob diameter = 0.5 μm), and signals that appear in consecutive frames within a distance of 500 nm were assigned to the same trajectory by the simple linear assignment problem tracker (linking max distance = 0.5 μm, gap-closing max distance = 0 μm, and gap-closing max frame gap = 0). Tracks containing at least 10 time lags were selected for HMM-Bayes analysis. In our case, >99% of tracks were identified by HMM-Bayes as purely diffusive tracks. The average diffusion (*D*_eff_), percentage of molecules that exhibit multiple diffusion states, and the average amplitude of motion (diffusion) switching (Δ*D*_eff_) between different states were calculated. The error associated with the HMM-Bayes fitting to the diffusion equation was ~15% on average, which was propagated to be 4.8 and 1.4% uncertainties in average *D*_eff_ for Gag in gRNA^+^ cells and gRNA^−^ cells, respectively.

### Fluorescence in situ hybridization

FISH of gRNA was performed following procedures similar to those previously described (*21*). Briefly, after cell fixation followed by incubation in 70% (v/v) ethanol at 4°C overnight, samples were washed thrice with wash buffer [10% (v/v) formamide in 2× saline sodium citrate (SSC)] and incubated in hybridization buffer [10% (w/v) dextran sulfate, 10% (v/v) formamide, and 2× SSC] containing 50 nM of a pool of 48 Quasar 670–labeled FISH probes that are complementary to different gRNA regions (*21*) at 37°C for 24 hours. Following three washes in wash buffer and then two washes in 2× SSC to remove the unbound probes, samples were incubated in 1× PBS for imaging.

### Colocalization analysis

The extent of colocalization between mEos3.1 signals and gRNA or gRNA mini FISH signals at the PM and in the particles (absorbed on poly-l-lysine–coated coverslips) was determined following procedures described previously (*19*, *20*). For the PM, a merged image of the mEos3.1 and FISH channels was first created using Fiji, and in the image, a region of interest around the PM was drawn to create a binary region of interest mask. Following application of this mask to both of the original mEos3.1 and FISH images, the local maxima of the mEos3.1 and FISH signals enclosed by the common mask were identified using the Find Maxima command available in Fiji. Colocalization events were determined on the basis of the local maxima of the isolated signals using a custom MATLAB program. Specifically, an mEos3.1 local maximum was treated as a colocalization event if an FISH local maximum was found within a 5 × 5–pixel square centered around the mEos3.1 maximum, and vice versa. The extent of Gag-mEos3.1 signals that colocalized with FISH signals (%Colocalization mEos3.1 at PM) was calculated by dividing the number of colocalization events by the total number of mEos3.1 local maxima. The percentage of FISH signals that colocalized with Gag-mEos3.1 signals (%Colocalization FISH at PM) was calculated by dividing the number of colocalization events by the total number of FISH local maxima. For particles, the local maxima in respective image were determined using the Find Maxima command available in Fiji. Each local maximum is considered an mEos3.1 or FISH-labeled particle. After determining the 2D coordinates of each particle in the respective channel, colocalization events, %Colocalization mEos3.1 in particles, and %Colocalization FISH in particles were determined on the basis of the local maxima as described above.

### Analysis of PM protein partitioning at Gag assembly sites

The degrees of PM protein partitioning at the Gag assembly sites were determined as described previously (*32*). Briefly, TIRF images of Gag-mCherry–labeled assembly sites were segmented to generate binary masks. These binary masks were then applied to the TIRF images of PM proteins to calculate average EGFP fluorescence intensities within the assembly sites (*I*_AS_). The degree of PM protein partitioning at a single Gag assembly site was calculated by dividing *I*_AS_ by the average EGFP intensity of the PM (*I*_PM_), with a value of >1 representing enrichment of PM proteins at the assembly site and a value of <1 representing depletion of PM proteins at the assembly site.

### Data analysis

All experiments were repeated at least three times. Unless otherwise noted, statistical analyses were performed using either two-tailed Student’s *t* test or one-way analysis of variance (ANOVA) with post hoc testing of pairwise comparisons using Dunnett’s T3 test using IBM SPSS Statistics, version 24. See table S5 for all *P* values.
