# Supplementary material for: Roles of RNA scaffolding in nanoscale Gag multimerization and selective protein sorting at HIV membranes
Source: Sci Adv. 2024 Feb 23;10(8):eadk8297. doi: 10.1126/sciadv.adk8297 (PMC10889351; doi:10.1126/sciadv.adk8297)
Supplement: Supplementary file 1 — Figs. S1 to S17 Tables S1 to S5 [file sciadv.adk8297_sm.pdf]

Science Advances
AAAS

Supplementary Materials for

Roles of RNA scaffolding in nanoscale Gag multimerization and selective protein sorting at HIV membranes

Yachen Ying *et al.*

Corresponding author: Antony K. Chen, chenak@pku.edu.cn

Sci. Adv. **10**, eadk8297 (2024)
DOI: 10.1126/sciadv.adk8297

This PDF file includes:

Figs. S1 to S17
Tables S1 to S5

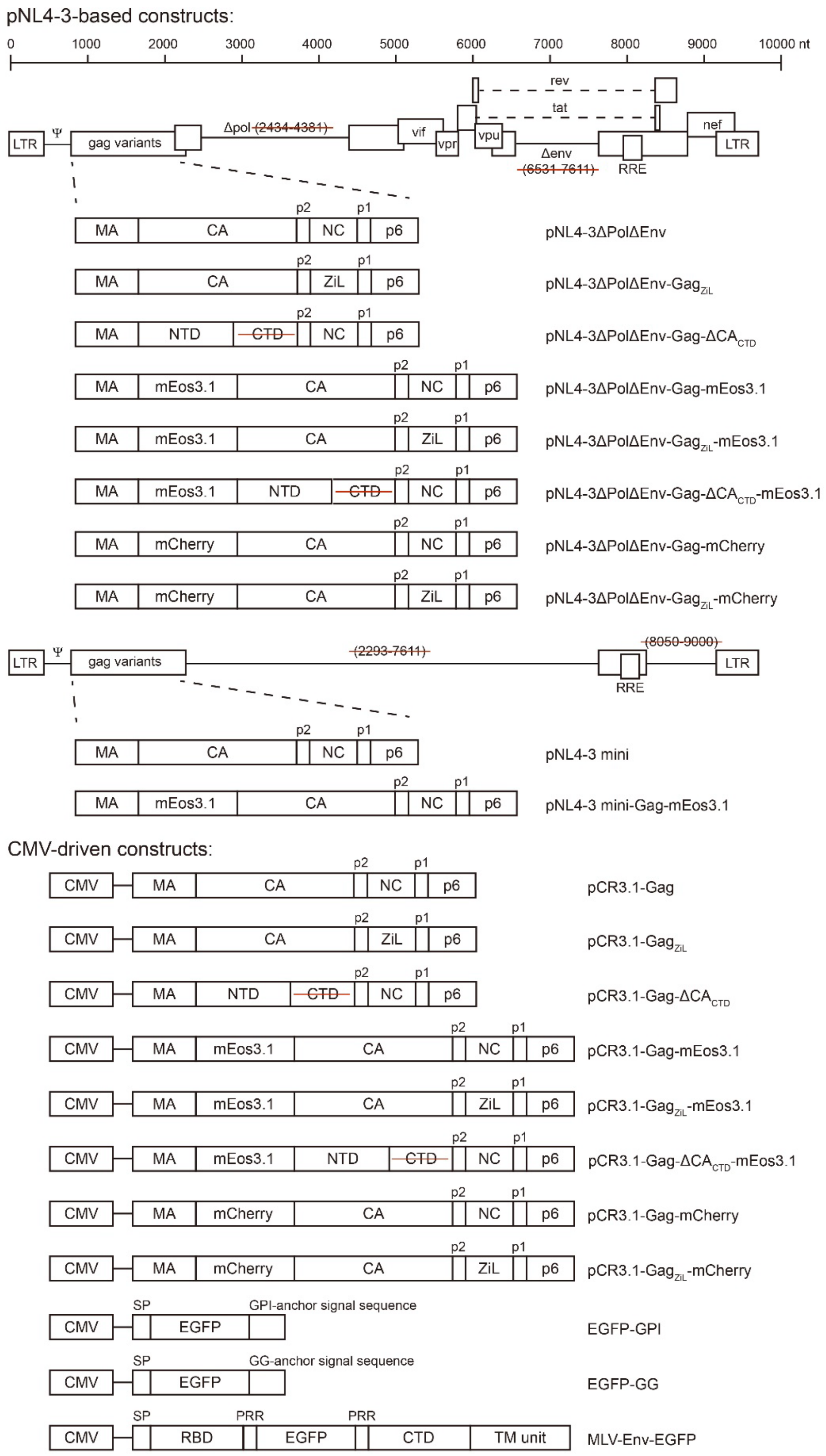

Fig. S1. Schematic representation of the pNL4-3-based and CMV-driven constructs used in this study.

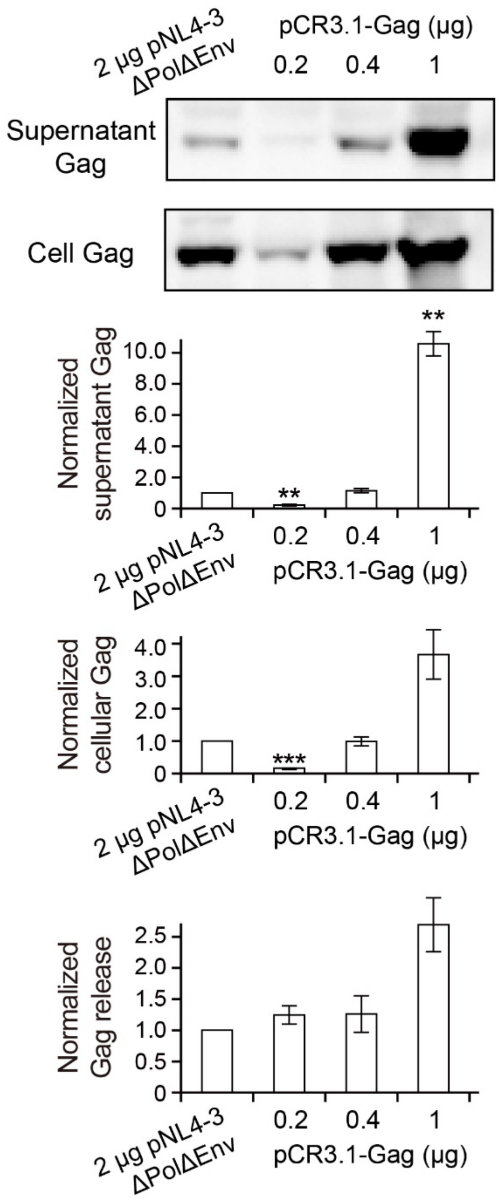

Fig. S2. Establishing a transfection protocol for pNL4-3-based and pCR3.1-based constructs that yield similar Gag expression levels and Gag release. Western blot analysis of cell and supernatant Gag at ~18 h post-transfection of 2 μg pNL4-3ΔPolΔEnv or pCR3.1-Gag at the indicated amounts. Data represent mean ± SEM of four experiments. Asterisks indicate significant differences from the 2 μg pNL4-3ΔPolΔEnv transfected sample (** $P < 0.01$, *** $P < 0.001$). Note that the pNL4-3ΔPolΔEnv sample and the 0.4 μg pCR3.1-Gag sample exhibited similar Gag levels in the cell and supernatant, and comparable Gag release. Also note that these transfection quantities could lead to similar Gag and Gag-mEos3.1 levels and comparable release when comparing cells transfected with pNL4-3ΔPolΔEnv-Gag-mEos3.1 and pCR3.1-Gag-mEos3.1 (co-transfected in a 1:10 ratio with the corresponding untagged constructs) (see Fig. 1).

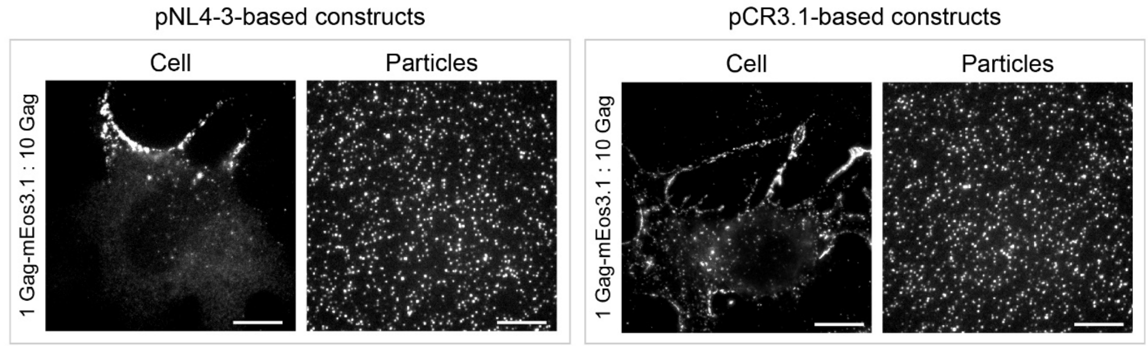

Fig. S3. Representative images of the Gag (detected by mEos3.1) in cells and in released particles. COS7 cells were transfected with pNL4-3ΔPolΔEnv-Gag-mEos3.1 or pCR3.1-Gag-mEos3.1 (co-transfected with the respective untagged construct in a 1:10 ratio). Note that the images merely show Gag-mEos3.1 expressed from either system can be incorporated into released particles and are not used for quantification. Scale bar, 10 μm.

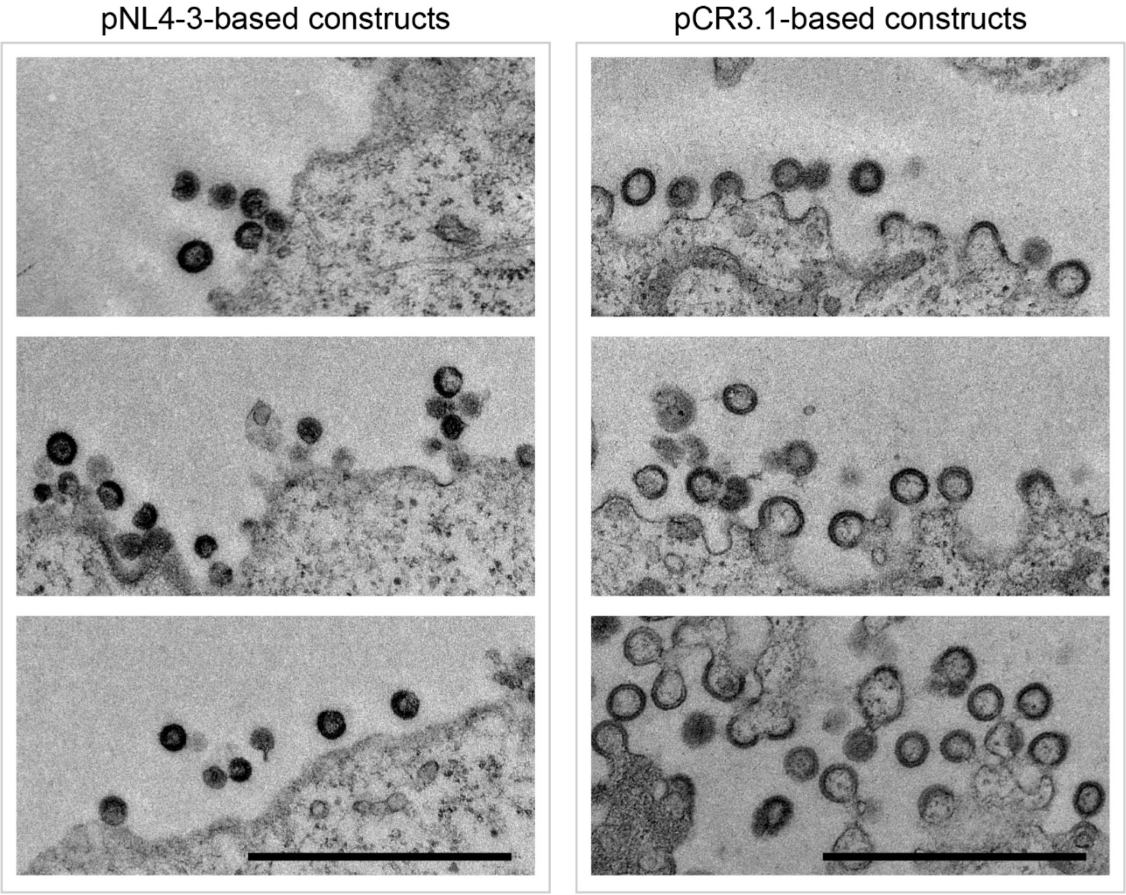

Fig. S4. Representative transmission electron microscopy images of assembling Gag particles at the PM of COS7 cells. Cells were transfected with pNL4-3ΔPolΔEnv-Gag-mEos3.1 or pCR3.1-Gag-mEos3.1 (co-transfected with the respective untagged construct in a 1:10 ratio). Scale bar, 1 μm.

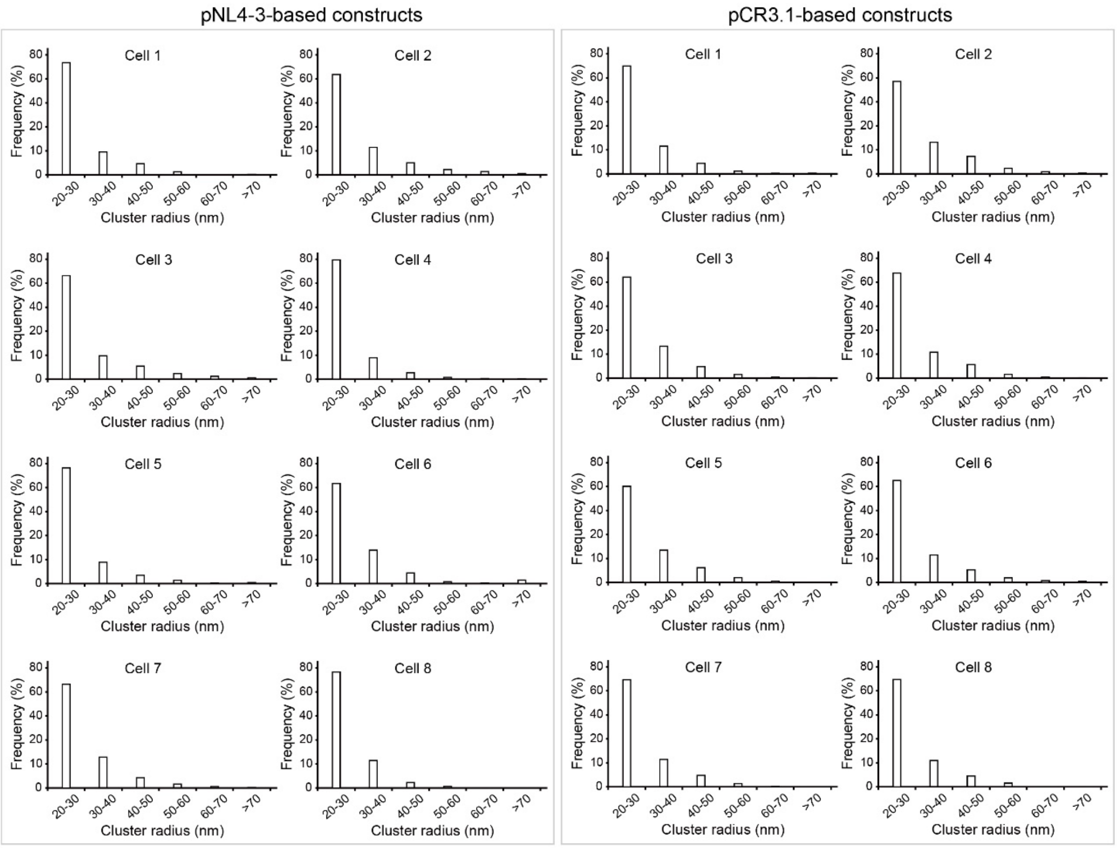

Fig. S5. Cluster radius distribution of Gag-mEos3.1 in each of the 8 gRNA$^+$ cells (COS7 cells co-transfected with pNL4-3ΔPolΔEnv-Gag-mEos3.1 in a 1:10 ratio with pNL4-3ΔPolΔEnv) and the 8 gRNA$^-$ cells (COS7 cells co-transfected with pCR3.1-Gag-mEos3.1 in a 1:10 ratio with pCR3.1-Gag) used in this study (See Fig. 2B). Note that the distributions are similar among cells under each transfection condition. This suggests that the 1:10 co-transfection/co-expression ratio was maintained across different cells.

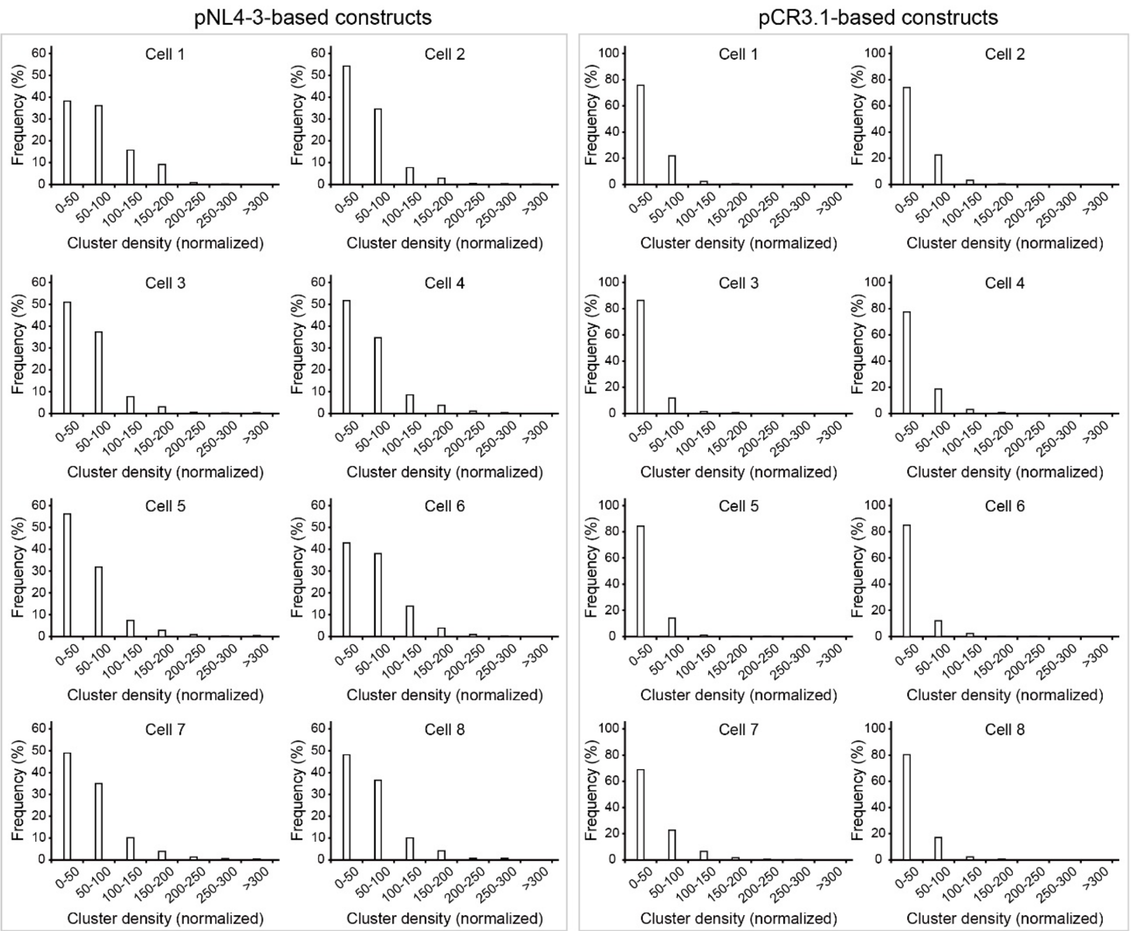

Fig. S6. Cluster density distribution of Gag-mEos3.1 in each of the 8 gRNA$^+$ cells (COS7 cells co-transfected with pNL4-3ΔPolΔEnv-Gag-mEos3.1 in a 1:10 ratio with pNL4-3ΔPolΔEnv) and the 8 gRNA$^-$ cells (COS7 cells co-transfected with pCR3.1-Gag-mEos3.1 in a 1:10 ratio with pCR3.1-Gag) used in this study (See Fig. 2C). Note that the distributions are similar among cells under each transfection condition. This suggests that the 1:10 co-transfection/co-expression ratio was maintained across different cells.

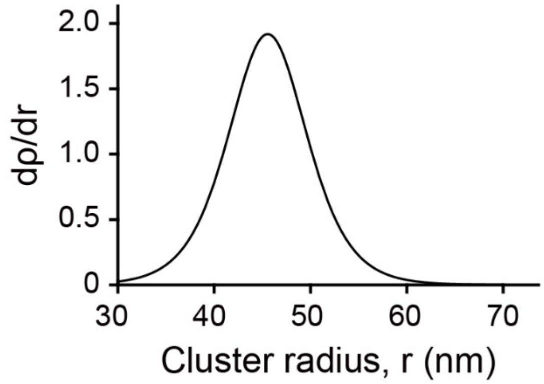

Fig. S7. Analysis of transition in cluster growth of Gag in gRNA$^+$ cells. The inflection point of cluster density as a function of cluster radius was determined by plotting the derivative of cluster density (ρ) versus cluster radius (r) (dρ/dr) as a function of cluster radius. The cluster radius that corresponds to the greatest change in ρ is considered the inflection point. The inflection point is approximately 45.5 nm for Gag in gRNA$^+$ cells.

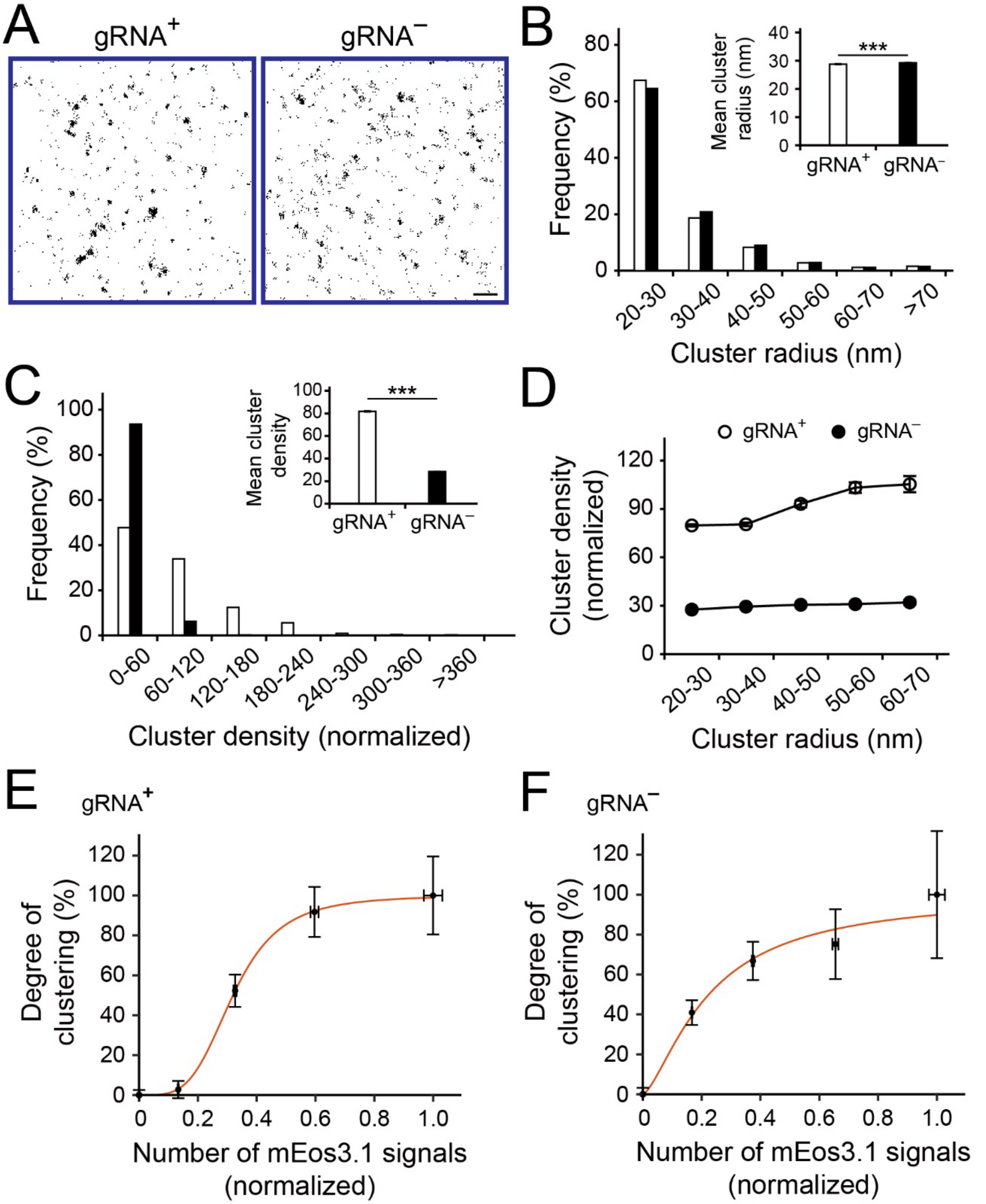

Fig. S8. Nanoscale organization of Gag at the PM of HeLa cells. HeLa cells were transfected with pNL4-3ΔPolΔEnv-Gag-mEos3.1 or pCR3.1-Gag-mEos3.1 (co-transfected with the respective untagged construct in a 1:10 ratio). **(A)** Representative PALM images of Gag in gRNA$^+$ and gRNA$^-$ cells. Individual spots represent single molecules. Scale bar, 500 nm. **(B)** Cluster radius distribution of Gag in gRNA$^+$ cells (n = 10,403 clusters from 8 cells) and gRNA$^-$ cells (n = 22,415 clusters from 8 cells). The inset shows mean ± SEM radius. **(C)** Cluster density distribution of Gag in gRNA$^+$ cells (n = 10,403 clusters from 8 cells) and gRNA$^-$ cells (n = 22,415 clusters from 8 cells). For each

cell, the cluster densities were normalized with respect to the mean density across the entire PM. The inset shows mean ± SEM density. **(D)** Gag cluster density of $gRNA^+$ and $gRNA^-$ cells from (C) plotted as a function of cluster radius. **(E-F)** Gag cluster density from (D) was further normalized with respect to the highest mean value, and the results (i.e., degree of clustering) were plotted as a function of the number of mEos3.1 signals detected within clusters for **(E)** $gRNA^+$ and **(F)** $gRNA^-$ cells. The red line represents the non-linear least-squares fitting of a four-parameter logistic regression model analogous to the Hill equation. For $gRNA^+$ cells, $r^2 = 0.9998$ and apparent cooperative index (n_H) = 3.96. For $gRNA^-$ cells, $r^2 = 0.9716$ and $n_H = 1.46$. For B-F, values were extracted from fixed-cell PALM images using a Hoshen-Kopelman-based algorithm as described in Methods. Asterisks indicate *P*-values (*** $P < 0.001$).

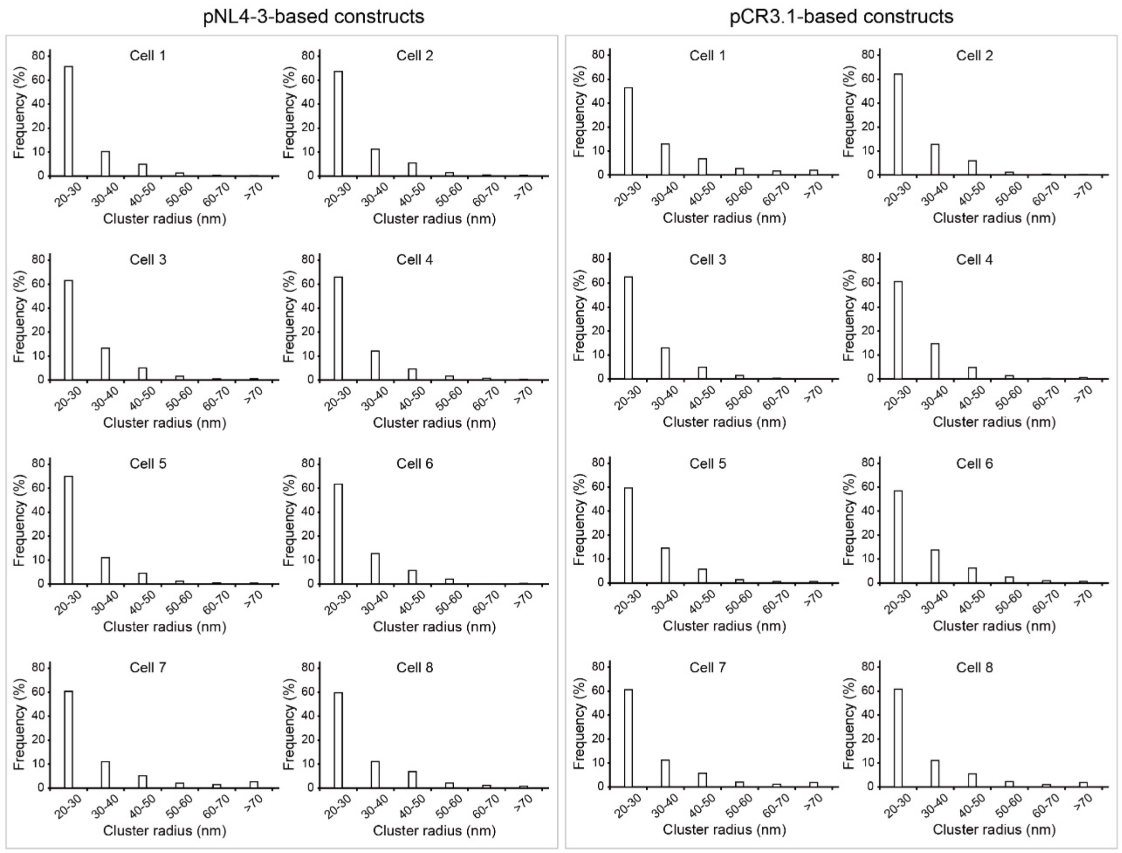

Fig. S9. Cluster radius distribution of Gag-mEos3.1 in each of the 8 gRNA⁺ cells (HeLa cells co-transfected with pNL4-3ΔPolΔEnv-Gag-mEos3.1 in a 1:10 ratio with pNL4-3ΔPolΔEnv) and the 8 gRNA⁻ cells (HeLa cells co-transfected with pCR3.1-Gag-mEos3.1 in a 1:10 ratio with pCR3.1-Gag) used in this study (See fig. S8B). Note that the distributions are similar among cells under each transfection condition. This suggests that the 1:10 co-transfection/co-expression ratio was maintained across different cells.

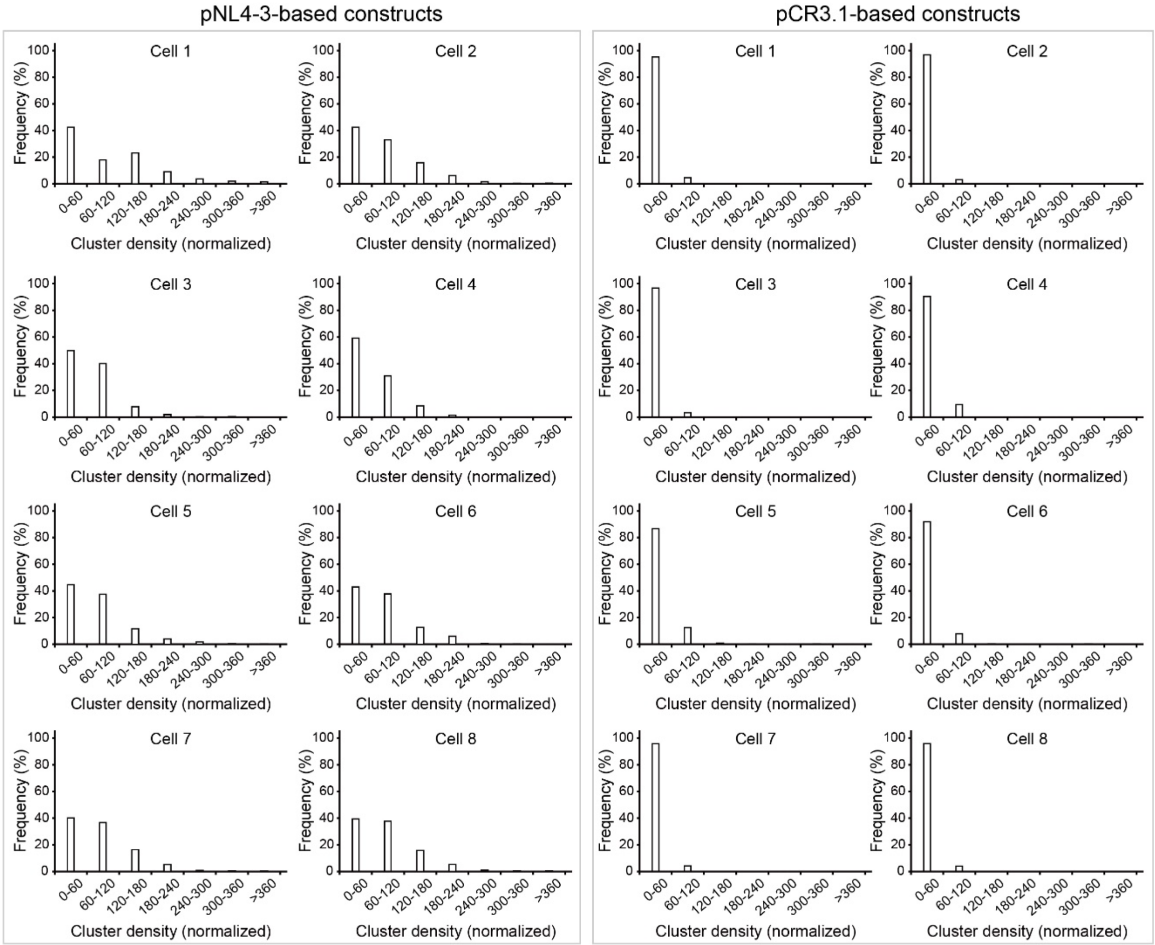

Fig. S10. Cluster density distribution of Gag-mEos3.1 in each of the 8 gRNA⁺ cells (HeLa cells co-transfected with pNL4-3ΔPolΔEnv-Gag-mEos3.1 in a 1:10 ratio with pNL4-3ΔPolΔEnv) and the 8 gRNA⁻ cells (HeLa cells co-transfected with pCR3.1-Gag-mEos3.1 in a 1:10 ratio with pCR3.1-Gag) used in this study (See fig. S8C). Note that the distributions are similar among cells under each transfection condition. This suggests that the 1:10 co-transfection/co-expression ratio was maintained across different cells.

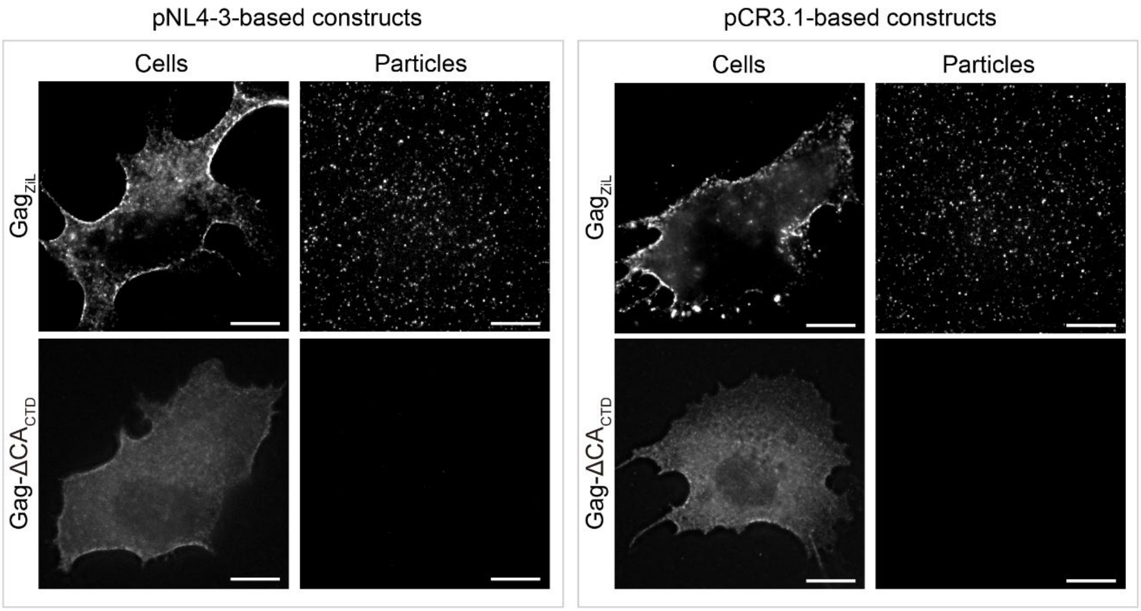

Fig. S11. Representative images of the Gag_{ZiL} and Gag-ΔCA_{CTD} (detected by mEos3.1) in cells and in released particles. COS7 cells were transfected with pNL4-3ΔPolΔEnv-Gag_{ZiL}-mEos3.1, pNL4-3ΔPolΔEnv-Gag-ΔCA_{CTD}-mEos3.1, pCR3.1-Gag_{ZiL}-mEos3.1 or pCR3.1-Gag-ΔCA_{CTD}-mEos3.1 (co-transfected with the respective untagged construct in a 1:10 ratio). Note that the images are not used for quantification. Scale bar, 10 μm.

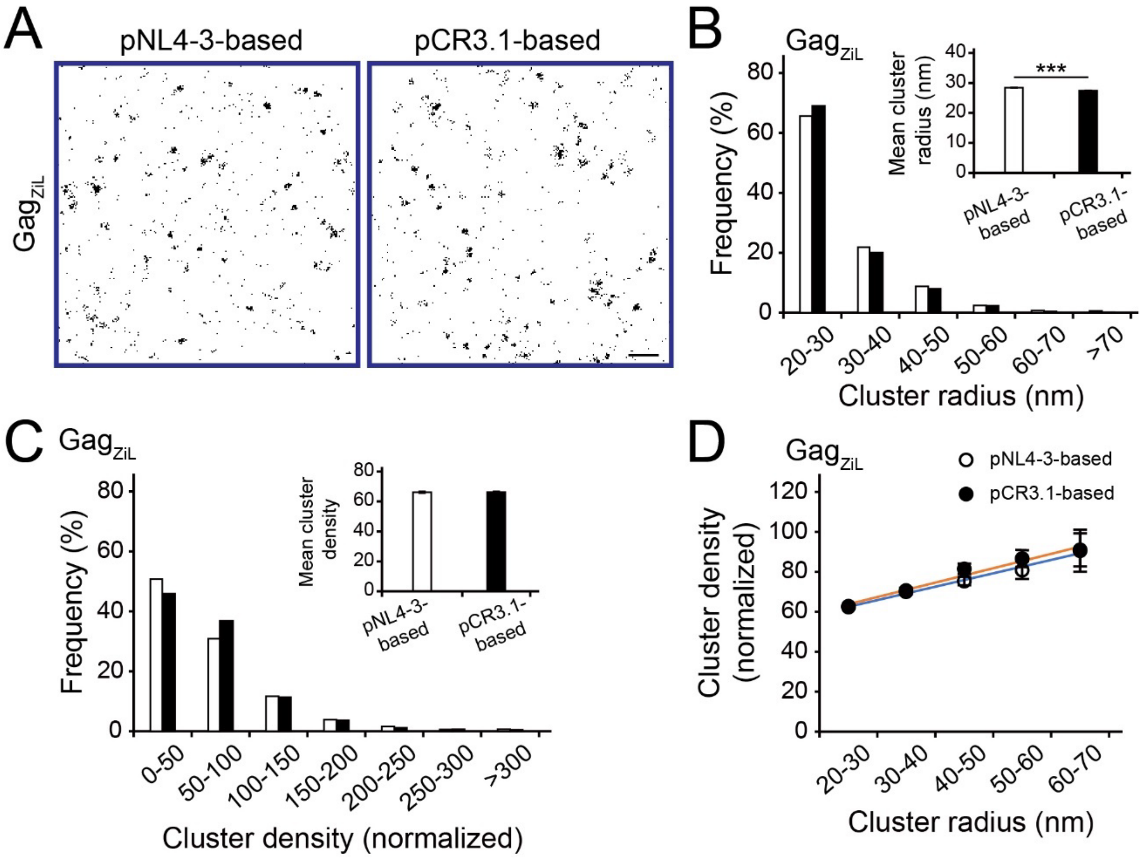

Fig. S12. Nanoscale organization of Gag_{ZiL} at the PM. COS7 cells were transfected with pNL4-3ΔPolΔEnv-Gag_{ZiL}-mEos3.1 or pCR3.1-Gag_{ZiL}-mEos3.1 (co-transfected with the respective untagged construct in a 1:10 ratio). **(A)** Representative PALM images of Gag_{ZiL} expressed from the pNL4-3-based and the pCR3.1-based constructs, respectively. Individual spots represent single molecules. Scale bar, 500 nm. **(B)** Cluster radius distribution of Gag_{ZiL} in cells transfected with the pNL4-3-based constructs (n = 9,042 clusters from 11 cells) and those with the pCR3.1-based constructs (n = 7,703 clusters from 8 cells). The inset shows mean ± SEM radius. **(C)** Cluster density distribution of Gag_{ZiL} in cells transfected with the pNL4-3-based constructs (n = 9,042 clusters from 11 cells) and those with the pCR3.1-based constructs (n = 7,703 clusters from 8 cells). For each cell, the cluster densities were normalized with respect to the mean density across the entire PM. The inset shows mean ± SEM density. **(D)** Gag_{ZiL} cluster density from (C) plotted as a function of cluster radius. The blue and orange lines represent the least square linear regressions for cells transfected with the pNL4-3-based constructs ($r^2 = 0.984$) and those with the pCR3.1-based constructs ($r^2 = 0.965$), respectively. For B-D, values were extracted from fixed-cell PALM images using a Hoshen-Kopelman-based algorithm. Asterisks indicate *P*-values (*** $P < 0.001$).

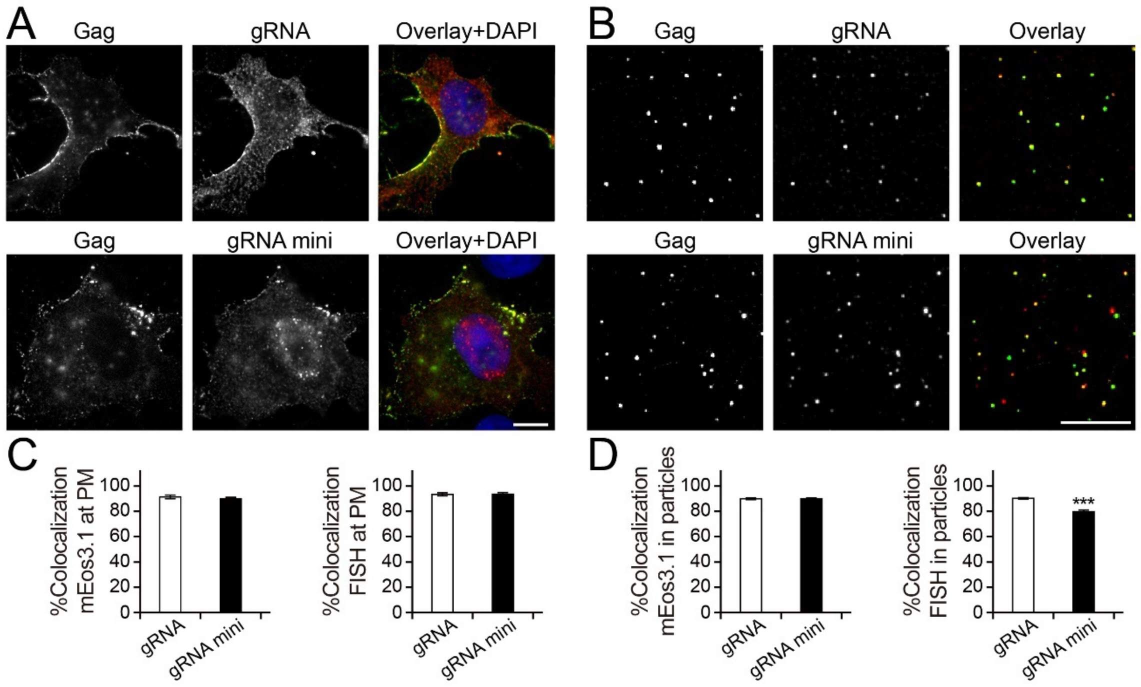

Fig. S13. Detection of Gag colocalization with gRNA versus gRNA mini at the cell PM and in released particles. For the analysis of Gag and gRNA colocalizations, COS7 cells were co-transfected with pNL4-3ΔPolΔEnv-Gag-mEos3.1 in a 1:10 ratio with pNL4-3ΔPolΔEnv (i.e., $gRNA^+$ cells). For the analysis of Gag and gRNA mini colocalizations, COS7 cells were co-transfected with pNL4-3 mini-Gag-mEos3.1 in a 1:10 ratio with pNL4-3 mini, along with Tat and Rev expressing helper constructs (See Methods). **(A)** Representative images of Gag (detected by mEos3.1) and gRNA or gRNA mini (detected by FISH probes) in cells. Scale bar, 10 µm. **(B)** Representative images of Gag (detected by mEos3.1) and gRNA or gRNA mini (detected by FISH probes) in particles. Scale bar, 10 µm. **(C)** Colocalization analysis between Gag and gRNA or gRNA mini at the PM of cells from (A). The percentage of mEos3.1 signals that were colocalized with FISH signals (%Colocalization mEos3.1 at PM) and the percentage of FISH signals that were colocalized with mEos3.1 signals (%Colocalization FISH at PM) were calculated. Data represent mean ± SEM of 25 $gRNA^+$ cells and 22 cells with gRNA mini. **(D)** Colocalization analysis between Gag and gRNA or gRNA mini in particles from (B). The percentage of mEos3.1 signals that were colocalized with FISH signals (%Colocalization mEos3.1 in particles) and the percentage of FISH signals that were colocalized with mEos3.1 signals (%Colocalization FISH in particles) were calculated. Data represent mean ± SEM of at least 20 independently acquired images for each experimental condition. Asterisks indicate P-values (*** $P < 0.001$).

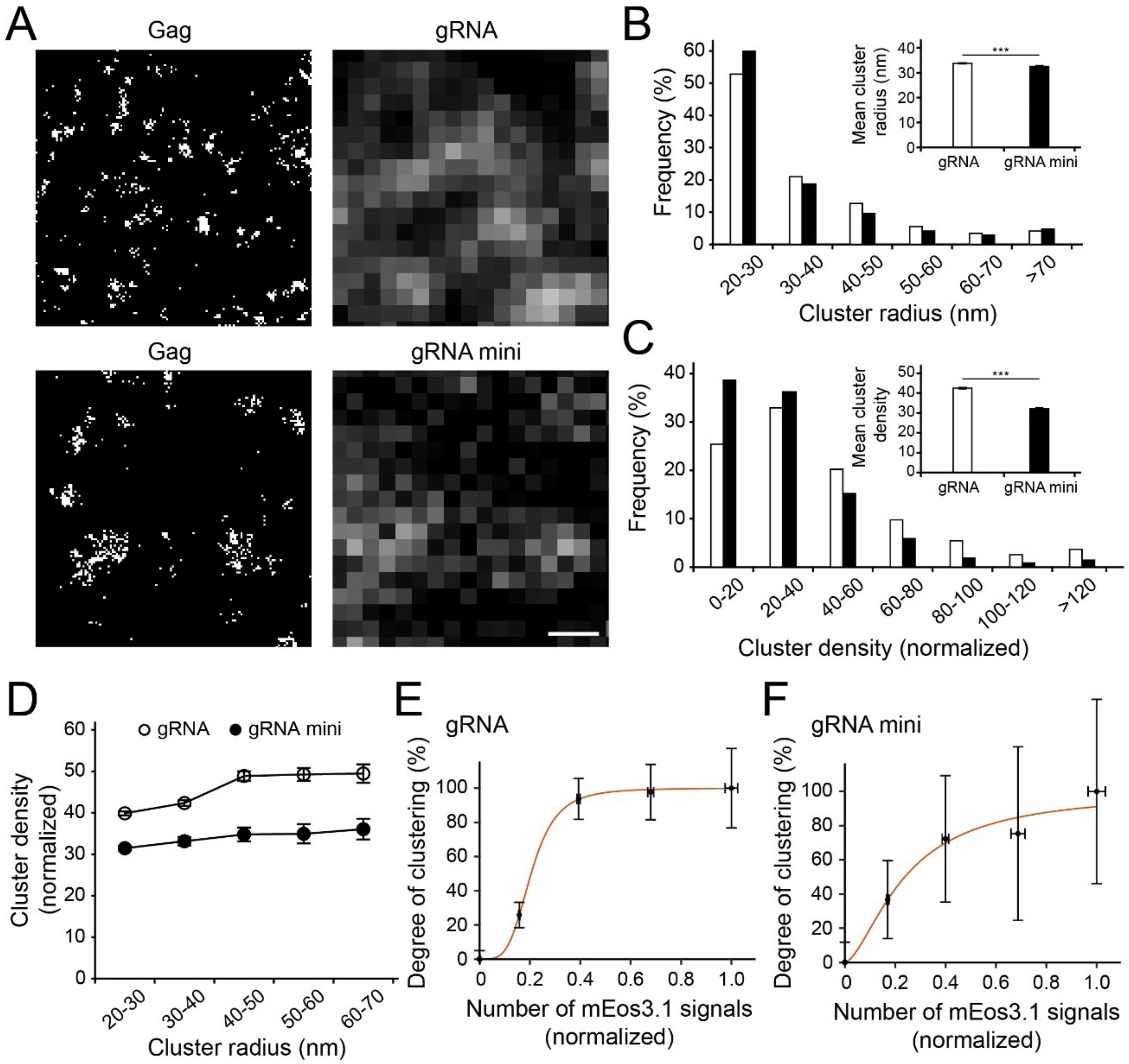

Fig. S14. Detection of nanoscale clustering of Gag around gRNA versus gRNA mini at the cell PM. For the analysis of nanoscale clustering of Gag around gRNA, COS7 cells were co-transfected with pNL4-3ΔPolΔEnv-Gag-mEos3.1 in a 1:10 ratio with pNL4-3ΔPolΔEnv (i.e., $gRNA^+$ cells). For the analysis of nanoscale clustering of Gag around gRNA mini, COS7 cells were co-transfected with pNL4-3 mini-Gag-mEos3.1 in a 1:10 ratio with pNL4-3 mini, along with Tat and Rev expressing helper constructs. Thereafter, samples were subjected to RNA FISH treatment to locate gRNA or gRNA mini. gRNA and gRNA mini were imaged via diffraction-limited TIRF imaging, whereas Gag was imaged by PALM. Note that the FISH images were used to generate gRNA and gRNA mini positive assembly platform masks for PALM cluster analysis of Gag (See Materials and Methods). **(A)** Representative PALM images of Gag-mEos3.1 and the corresponding diffraction-limited images of gRNA and gRNA mini (detected by FISH probes) at the PM of $gRNA^+$ cells and cells expressing gRNA mini, respectively. Scale bar, 500 nm. **(B)** Cluster radius distribution in $gRNA^+$ cells (n = 8,777 clusters from 9 cells) and cells expressing gRNA mini (n = 3,220 clusters from 9 cells). The inset shows mean ± SEM radius. **(C)** Cluster density distribution of Gag in $gRNA^+$ cells (n = 8,777 clusters from 9 cells) and cells expressing gRNA mini (n = 3,220 clusters from 9 cells). For each cell, the

cluster densities were normalized with respect to the mean density across the entire PM. The inset shows mean ± SEM density. **(D)** Gag cluster density from (C) plotted as a function of cluster radius. **(E-F)** Gag cluster density from (D) was further normalized with respect to the highest mean value, and the results (i.e., degree of clustering) were plotted as a function of the number of mEos3.1 signals detected within clusters for **(E)** $gRNA^+$ cells and **(F)** cells expressing gRNA mini. The red line represents the non-linear least-squares fitting of a four-parameter logistic regression model analogous to the Hill equation. For $gRNA^+$ cells, r^2 = 0.9997 and apparent cooperative index (n_H) = 4.07. For cells expressing gRNA mini, r^2 = 0.9713 and n_H = 1.62. For B-F, values were extracted from fixed-cell PALM images using a Hoshen-Kopelman-based algorithm as described in Materials and Methods. Asterisks indicate *P*-values (*** $P < 0.001$).

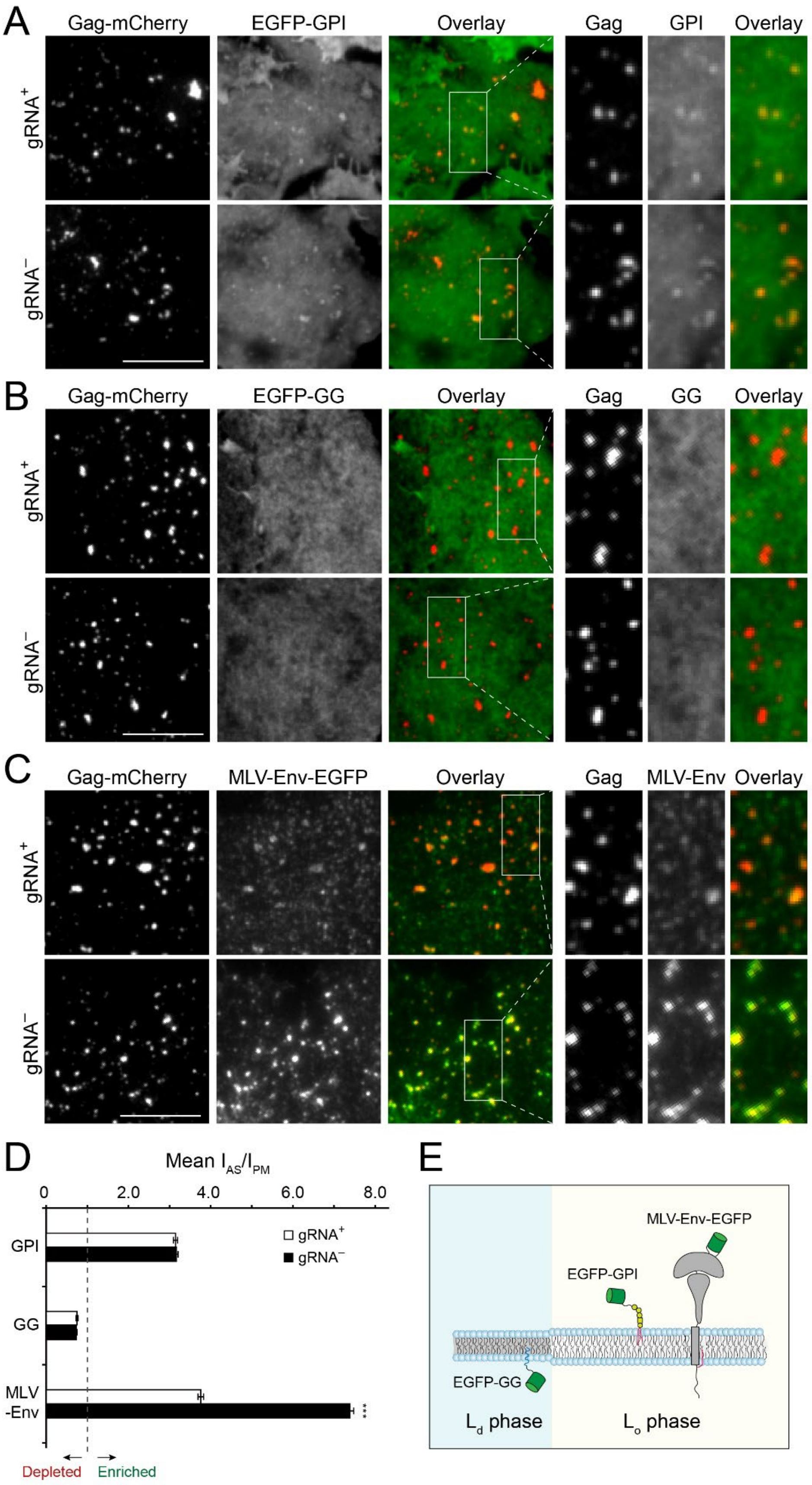
A
Gag-mCherry
EGFP-GPI
Overlay
Gag
GPI
Overlay
gRNA+
gRNA−
B
Gag-mCherry
EGFP-GG
Overlay
Gag
GG
Overlay
gRNA+
gRNA−
C
Gag-mCherry
MLV-Env-EGFP
Overlay
Gag
MLV-Env
Overlay
gRNA+
gRNA−
D
Mean I_AS/I_PM
0
2.0
4.0
6.0
8.0
GPI
GG
MLV
-Env
□ gRNA+
■ gRNA−
***
Depleted
Enriched
E
MLV-Env-EGFP
EGFP-GPI
EGFP-GG
Ld phase
Lo phase

Fig. S15. Differential PM protein partitioning into cellular RNA-mediated versus gRNA-mediated Gag assembly sites in HeLa cells. HeLa cells stably expressing EGFP-GPI, EGFP-GG and MLV-Env-EGFP were co-transfected with pNL4-3ΔPolΔEnv-Gag-mCherry or pCR3.1-Gag-mCherry in a 1:10 ratio with the respective untagged construct. **(A-C)** Representative TIRF images of Gag-mCherry and **(A)** EGFP-GPI, **(B)** EGFP-GG, and **(C)** MLV-Env-EGFP at the PM of $gRNA^+$ and $gRNA^−$ cells at ~18 h post-transfection. The panels on the right are magnified images of the boxed areas in the images. Scale bar, 10 μm. **(D)** Extents of enrichment and depletion of indicated PM proteins at the Gag assembly sites (Mean ± SEM I_{AS}/I_{PM}) in $gRNA^+$ and $gRNA^−$ cells (see Methods). For EGFP-GPI, n = 1,559 assembly sites from 29 $gRNA^+$ cells and n = 2,201 assembly sites from 25 $gRNA^−$ cells. For EGFP-GG, n = 1,720 assembly sites from 17 $gRNA^+$ cells and n = 1,793 assembly sites from 18 $gRNA^−$ cells. For MLV-Env-EGFP, n = 1,563 assembly sites from 21 $gRNA^+$ cells and n = 3,155 assembly sites from 30 $gRNA^−$ cells. Asterisks indicate *P*-values (*** $P < 0.001$). **(E)** Schematic showing the lipid phase partitioning preferences of the EGFP fusion proteins.

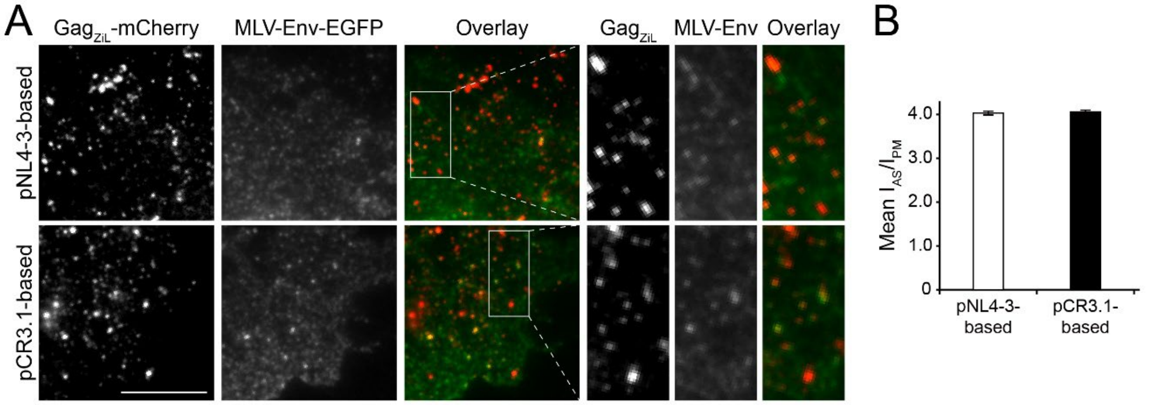

Fig. S16. Partitioning of MLV-Env-EGFP into Gag$_{ZiL}$-mediated assembly sites at the PM of COS7 cells. COS7 cells were transfected with MLV-Env-EGFP and pNL4-3ΔPolΔEnv-Gag$_{ZiL}$-mCherry or pCR3.1-Gag$_{ZiL}$-mCherry (Gag$_{ZiL}$ was co-transfected in a 1:10 ratio with the respective untagged construct). **(A)** Representative TIRF images of Gag$_{ZiL}$-mCherry and MLV-Env-EGFP at the PM of cells transfected with the pNL4-3-based or the pCR3.1-based constructs. The panels on the right are magnified images of the boxed areas in the images. Scale bar, 10 µm. **(B)** The degrees of enrichment of MLV-Env at the Gag$_{ZiL}$ assembly sites (Mean ± SEM I_{AS}/I_{PM}) in cells transfected with the pNL4-3-based constructs (n =5,569 assembly sites from 33 cells) and cells transfected with the pCR3.1-based constructs (n = 5,999 assembly sites from 31 cells).

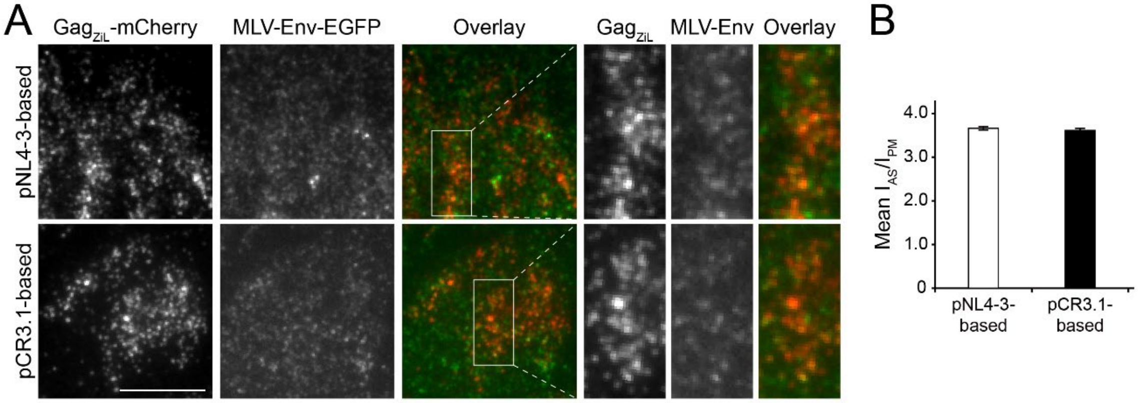

Fig. S17. Partitioning of MLV-Env-EGFP into Gag$_{ZiL}$-mediated assembly sites at the PM of HeLa cells. HeLa cells stably expressing MLV-Env-EGFP were co-transfected with pNL4-3ΔPolΔEnv-Gag$_{ZiL}$-mCherry or pCR3.1-Gag$_{ZiL}$-mCherry in a 1:10 ratio with the respective untagged construct. **(A)** Representative TIRF images of Gag$_{ZiL}$-mCherry and MLV-Env-EGFP at the PM of cells transfected with the pNL4-3-based or the pCR3.1-based constructs. The panels on the right are magnified images of the boxed areas in the images. Scale bar, 10 μm. **(B)** The degrees of enrichment of MLV-Env at the Gag$_{ZiL}$ assembly sites (Mean ± SEM I_{AS}/I_{PM}) in cells transfected with the pNL4-3-based constructs (n =3,573 assembly sites from 29 cells) and cells transfected with the pCR3.1-based constructs (n = 3,839 assembly sites from 25 cells).

Table S1. The quantity and normalized Gag density of Gag clusters detected across different cluster radius ranges in $gRNA^+$ and $gRNA^-$ cells from Fig. 2D.

Cluster radius (nm)	Number of clusters			Normalized cluster density		
	$gRNA^+$	$gRNA^-$	Ratio ($gRNA^+/gRNA^-$)	$gRNA^+$	$gRNA^-$	Ratio ($gRNA^+/gRNA^-$)
20-30	5795	8748	0.66	60.93	34.66	1.76
30-40	1381	2436	0.57	63.22	39.76	1.59
40-50	513	1079	0.48	78.35	41.20	1.90
50-60	174	342	0.51	81.61	42.21	1.93
60-70	63	108	0.58	81.74	40.69	2.01

Table S2. The sequences of PCR primers used for generating pNL4-3-based constructs

Construct	PCR primer sequence (5’ to 3’)	Template
pNL4-3ΔPolΔEnv-Gag-ΔCA$_{CTD}$	ACTCGGCTTGCTGAAGCGCGCACGG	pNL4-3ΔPolΔEnv
	TGGATTTGTTACTTGGCTCATTGCTTCAGCGCTATACATTCTTACTATTTTATTTAA	
	TTAAATAAAATAGTAAGAATGTATAGCGCTGAAGCAATGAGCCAAGTAACAAATCCA	
	GTTGCAGAATTCTTATTATGGCTTC	
pNL4-3ΔPolΔEnv-Gag-mEos3.1	AAGCCAGAGGAGATCTCTCGACGCA	pNL4-3ΔPolΔEnv
	AACGAATTGTACAATTGGATAGTTCTGCGAGACCTGGCTGTTGTTTCCTGTGTCA	
	CGATTAAGCCAAAATTACCCTATAGTGCAGA	
	CTTGTGAAGCTTGCTCGGCTCTTAG	
	TCGCAGAACTATCCAATTGTACAATTCGTTATGAGTGCGATTAAGCCAGA	mEos3.1-N1
	CTGCACTATAGGGTAATTTTGGCTTAATCGCTTGTACAGTCGTCTGGCATTGTCA	
pNL4-3 mini & pNL4-3 mini-Gag-mEos3.1	CAGGCCAGATGAGAGAACCAAGGGGA	pNL4-3ΔPolΔEnv
	CTCATATCGCCTCCTCCAGGTCTGAAGATCTTATTGTGACGAGGGGTCGCTGCCA	
	GATCTTCAGACCTGGAGGAGGCGATATG	
	TGTAAGTCATTGGTCTTAAAGGTACCTGAGGCATTCCAAGGCACAGCAGTGGTGC	
	CTCAGGTACCTTTAAGACCAATGACTTA	
	AGACCCTGCACTCCATGGATCAGCT	
pNL4-3ΔPolΔEnv-Gag-mCherry	AAGCCAGAGGAGATCTCTCGACGCA	pNL4-3ΔPolΔEnv
	AACGAATTGTACAATTGGATAGTTCTGCGAGACCTGGCTGTTGTTTCCTGTGTCA	
	CGATTAAGCCAAAATTACCCTATAGTGCAGA	
	CTTGTGAAGCTTGCTCGGCTCTTAG	
	TCGCAGAACTATCCAATTGTACAATTCGTTATGGTGAGCAAGGGCGAGGAGGATA	pmCherry-N1
	CTGCACTATAGGGTAATTTTGGCTTAATCGCTTGTACAGCTCGTCCATGCCGCCG	

Table S3. The sequences of PCR primers used for generating pCR3.1-based constructs that encode Gag and Gag variants

Construct	PCR primer sequence (5’ to 3’)	Template
pCR3.1-Gag$_{ZiL}$	CTGCAGCGTATGAAACAGATCGAAGACAAAATCGA	pNL4-3ΔPolΔEnv-Gag$_{ZiL}$
	CTCACCGATCAGTTTTTGATACGAGCGATTTCGT	
	AGACAGGCTAATTTTTTAGGGAAGATCTGG	pCR3.1-Gag
	CATGATGGTAGCGGAGTTGGTCACCTGGCT	
pCR3.1-Gag-mEos3.1	GCTGGCTAGCGTTTAAACTTAAGCT	pCR3.1-Gag
	AACGAATTGTACAATTGGATAGTTCTGCGAGACCTGGTTGCTGTGTCCGGTGTCC	
	CGATTAAGCCAGAACTACCCCATCGTGCAGA	
	CAGGCGGTCATCATTTCCTCTAGGGTA	
	TCGCAGAACTATCCAATTGTACAATTCGTTATGAGTGCGATTAAGCCAGACATGA	mEos3.1-N1
	CTGCACGATGGGGTAGTTCTGGCTTAATCGCTTGTACAGTCGTCTGGCATTGTCA	
pCR3.1-Gag-ΔCA$_{CTD}$	GAAGTACAAGCTGAAGCACATCGTG	pCR3.1-Gag
	GCTATACATGCGCACGATCTTGTTC	
	GGCCTGAACAAGATCGTGCGCATGTATAGCGCTGAGGCCATGAGCCAGGT	
	GCCCTCTAGACTCGAGAGATCCTTA	
pCR3.1-Gag-ΔCA$_{CTD}$-mEos3.1	GAAGTACAAGCTGAAGCACATCGTG	pCR3.1-Gag-mEos3.1
	GCTATACATGCGCACGATCTTGTTC	
	GGCCTGAACAAGATCGTGCGCATGTATAGCGCTGAGGCCATGAGCCAGGT	
	GCCCTCTAGACTCGAGAGATCCTTA	
pCR3.1-Gag-mCherry	GCTGGCTAGCGTTTAAACTTAAGCT	pCR3.1-Gag
	AACGAATTGTACAATTGGATAGTTCTGCGAGACCTGGTTGCTGTGTCCGGTGTCC	
	CGATTAAGCCAGAACTACCCCATCGTGCAGA	
	TTCTGGACCAGCAGGGTTTCGGT	
	TCGCAGAACTATCCAATTGTACAATTCGTTATGGTGAGCAAGGGCGAGGAGGATA	pmCherry-N1
	CTGCACGATGGGGTAGTTCTGGCTTAATCGCTTGTACAGCTCGTCCATGCCGCCG	

Table S4. The sequences of PCR primers used for generating constructs that encode EGFP-labeled PM proteins

Construct	PCR primer sequence (5’ to 3’)	Template
EGFP-GG	TAGTGAACCGTCAGATCCGCTAGCG	pEGFP-C1
	GCTGACACTTTGTCTTTGACTTCTTTTTCTTCTTTTTACCATCAGATCTGAGTCCGGACTTGTACAGCT	
	AGAAAAAGAAGTCAAAGACAAAGTGTCAGCTGCTTTAATAGCTGCAGTCGACGGTACCGCGGG	
	GCTGCAATAAACAAGTTAACAACAACAATTGC	

Table S5. The resulting *P*-values of statistical analyses performed in this work.

Figure	Description	*P*-value
Fig. 1A	Normalized Gag level in the cell (two-tailed Student's t-test)	0.47
Fig. 1B	Normalized Gag level in the supernatant (two-tailed Student's t-test)	0.81
Fig. 1C	Normalized Gag release (two-tailed Student's t-test)	0.69
Fig. 2B	Mean Gag cluster radius (two-tailed Student's t-test)	5.1×10^{-6}
Fig. 2C	Mean Gag cluster density (two-tailed Student's t-test)	0
Fig. 3B	Mean diffusion coefficient (two-tailed Student's t-test)	4.2×10^{-29}
Fig. 3C	Mean magnitude of motion switching (ΔD_{eff}) (two-tailed Student's t-test)	2.5×10^{-4}
Fig. 3F	Percentage of Gag clusters having uniform tcPALM profiles (two-tailed Student's t-test)	6.8×10^{-9}
	Percentage of Gag clusters having stepwise tcPALM profiles (two-tailed Student's t-test)	6.8×10^{-9}
Fig. 4D	Extend of enrichment of EGFP-GPI (two-tailed Student's t-test)	0.82
	Extend of depletion of EGFP-GG (two-tailed Student's t-test)	0.62
	Extend of enrichment of MLV-Env-EGFP (two-tailed Student's t-test)	0
fig. S2	Normalized supernatant Gag level (one-way ANOVA with post hoc testing of pairwise comparisons using Dunnett's T3 test), 2 µg of pNL4-3ΔPolΔEnv versus indicated amounts of pCR3.1-Gag	4.9×10^{-3} (0.2 µg of pCR3.1-Gag)
		0.89 (0.4 µg of pCR3.1-Gag)
		6.9×10^{-3} (1 µg of pCR3.1-Gag)
	Normalized cellular Gag level (one-way ANOVA with post hoc testing of pairwise comparisons using Dunnett's T3 test), 2 µg of pNL4-3ΔPolΔEnv versus indicated amounts of pCR3.1-Gag	2.4×10^{-4} (0.2 µg of pCR3.1-Gag)
		1 (0.4 µg of pCR3.1-Gag)
		0.19 (1 µg of pCR3.1-Gag)
	Normalized cellular Gag release (one-way ANOVA with post hoc testing of pairwise comparisons using Dunnett's T3 test), 2 µg of pNL4-3ΔPolΔEnv versus indicated amounts of pCR3.1-Gag	0.66 (0.2 µg of pCR3.1-Gag)
		0.95 (0.4 µg of pCR3.1-Gag)
		0.15 (1 µg of pCR3.1-Gag)
fig. S8B	Mean Gag cluster radius (HeLa) (two-tailed Student's t-test)	9.3×10^{-4}
fig. S8C	Mean Gag cluster density (HeLa) (two-tailed Student's t-test)	0
fig. S12B	Mean Gag_{ZiL} cluster radius (two-tailed Student's t-test)	5.3×10^{-11}
fig. S12C	Mean Gag_{ZiL} cluster density (two-tailed Student's t-test)	0.92
fig. S13C	%Colocalization mEos3.1 at PM (two-tailed Student's t-test)	0.44

	%Colocalization FISH at PM (two-tailed Student's t-test)	0.94
fig. S13D	%Colocalization mEos3.1 in particle (two-tailed Student's t-test)	0.94
	%Colocalization FISH in particle (two-tailed Student's t-test)	4.4×10^{-11}
fig. S14B	Mean Gag cluster radius (two-tailed Student's t-test)	3.5×10^{-4}
fig. S14C	Mean Gag cluster density (two-tailed Student's t-test)	9.3×10^{-70}
fig. S15D	Extend of enrichment of EGFP-GPI (HeLa) (two-tailed Student's t-test)	0.75
	Extend of depletion of EGFP-GG (HeLa) (two-tailed Student's t-test)	0.54
	Extend of enrichment of MLV-Env-EGFP (HeLa) (two-tailed Student's t-test)	3.8×10^{-196}
fig. S16B	Extend of enrichment of MLV-Env-EGFP (Gag_{ZiL}) (two-tailed Student's t-test)	0.65
fig. S17B	Extend of enrichment of MLV-Env-EGFP (HeLa, Gag_{ZiL}) (two-tailed Student's t-test)	0.43
